# An Adaption Broadcast Radius-Based Code Dissemination Scheme for Low Energy Wireless Sensor Networks

**DOI:** 10.3390/s18051509

**Published:** 2018-05-10

**Authors:** Shidi Yu, Xiao Liu, Anfeng Liu, Naixue Xiong, Zhiping Cai, Tian Wang

**Affiliations:** 1 School of Information Science and Engineering, Central South University, Changsha 410083, China; 0918150224@csu.edu.cn (S.Y.); afengliu@mail.csu.edu.cn (A.L.); 2 The State Key Laboratory of Industrial Control Technology, Zhejiang University, Hangzhou 310027, China; 3 Department of Mathematics and Computer Science, Northeastern State University, Tahlequah, OK 74464, USA; xiongnaixue@gmail.com; 4 Department of Network Engineering, School of Computer, National University of Defense Technology, Changsha 410073, China; zpcai@nudt.edu.cn; 5 School of Computer Science, National Huaqiao University, Quanzhou 362000, China; wangtian@hqu.edu.cn

**Keywords:** wireless sensor networks, energy efficiency, codes dissemination, minimum-transmission broadcast, delay

## Abstract

Due to the Software Defined Network (SDN) technology, Wireless Sensor Networks (WSNs) are getting wider application prospects for sensor nodes that can get new functions after updating program codes. The issue of disseminating program codes to every node in the network with minimum delay and energy consumption have been formulated and investigated in the literature. The minimum-transmission broadcast (MTB) problem, which aims to reduce broadcast redundancy, has been well studied in WSNs where the broadcast radius is assumed to be fixed in the whole network. In this paper, an Adaption Broadcast Radius-based Code Dissemination (ABRCD) scheme is proposed to reduce delay and improve energy efficiency in duty cycle-based WSNs. In the ABCRD scheme, a larger broadcast radius is set in areas with more energy left, generating more optimized performance than previous schemes. Thus: (1) with a larger broadcast radius, program codes can reach the edge of network from the source in fewer hops, decreasing the number of broadcasts and at the same time, delay. (2) As the ABRCD scheme adopts a larger broadcast radius for some nodes, program codes can be transmitted to more nodes in one broadcast transmission, diminishing the number of broadcasts. (3) The larger radius in the ABRCD scheme causes more energy consumption of some transmitting nodes, but radius enlarging is only conducted in areas with an energy surplus, and energy consumption in the hot-spots can be reduced instead due to some nodes transmitting data directly to sink without forwarding by nodes in the original hot-spot, thus energy consumption can almost reach a balance and network lifetime can be prolonged. The proposed ABRCD scheme first assigns a broadcast radius, which doesn’t affect the network lifetime, to nodes having different distance to the code source, then provides an algorithm to construct a broadcast backbone. In the end, a comprehensive performance analysis and simulation result shows that the proposed ABRCD scheme shows better performance in different broadcast situations. Compared to previous schemes, the transmission delay is reduced by 41.11~78.42%, the number of broadcasts is reduced by 36.18~94.27% and the energy utilization ratio is improved up to 583.42%, while the network lifetime can be prolonged up to 274.99%.

## 1. Introduction

Wireless sensor networks (WSNs) which are composed of inexpensive microprocessors, with the ability of wireless communication, computing, storage and sensing the surrounding environment [1,2,3,4,5], are emerging as promising platforms that enable a wide range of applications in numerous application areas such as smart cities [6], traffic monitoring [7], automation control in factories [8,9], monitoring public facilities, the environment, or weather [10,11,12,13], human health monitoring [14], wildlife protection, military applications and so on [15,16,17,18]. With the development of micro-processing technology, sensor nodes are becoming more and more powerful, while their volume is getting smaller and smaller, the price is getting lower and lower, and their application prospects are becoming more and more widespread [19,20,21,22,23]. One of the most important technologies that makes WSNs’ vitality grow is Software Defined Network (SDN) technology [24,25,26]. SDN technology is an idea of replacing hardware with software for designing. In this way, mainly through updating the software of the network equipment, the network equipment can extend its original functionality, or acquire new functions to adapt to a new application environment [24,25,26]. SDN technology is fast updating, low-cost, flexible, and adaptable for large-scale applications, attracting extensive attention from researchers and industry [24,25,26]. Among many related research issues, an important one is how to disseminate program codes to all nodes in the network quickly and with low energy consumption, so that the software of these nodes can be updated to have new or additional functions [25]. For example, in an industrial sensor network, the monitoring of an industrial site needs the collected image acquisition accuracy to be further improved. By updating the sensor node program code, the accuracy of the collected data is improved, so that we can better meet the needs of industrial site monitoring [25]. However, updating program codes is not an easy task, as it is subject to the following restrictions: first, program codes need to be disseminated to every node in the network as quickly as possible, because, during the replacement of program codes, there may be inconsistencies in the format and function of the data collected by the nodes in the network, which may affect the decisions made by the system. This inconsistency may bring serious losses to the industrial control system. Thus, that time when any inconsistency between nodes may exist should be as short as possible. That is, the time taken for disseminating program codes should be as short as possible [25], but in order to save energy, sensor nodes often adopt an alternatively sleep/awake duty-cycle way of working which brings more delay for codes dissemination [27,28], because while nodes are in sleep status, their energy consumption is only 0.1~1% of that in an awake status, but the nodes can’t transmit, receive and sense data in sleep status. Thus, much delay is generated for code dissemination. Next, code dissemination needs to use as little energy as possible. Sensor nodes are generally battery-powered and therefore have limited energy [29,30,31,32,33], and the sensor network deployment environment is generally dangerous [34,35,36,37,38], or other they are in other restricted environments, so battery replacement is precluded after deployment [31,39,40]. Therefore, the process of program code dissemination must save energy as far as possible, to prolong network lifetime [24,27,28]. Since energy consumption is positively related to the number of broadcasts of the codes, the key to reducing energy consumption is how to reduce the number of broadcasts [28]. Researchers have summarized this issue as a minimum-transmission broadcast (MTB) problem [27,28]. Le Duc et al. [27] point out that the MTB problem in duty-cycled networks (MTB-DC problem) is proved to be NP-hard, and it is still a challenge to design another algorithm which has better results to further reduce the number of transmissions. In general, reducing the number of transmissions will reduce the energy consumption of the network, but reducing the number of transmissions will not necessarily improve the network lifetime and reduce the code transmission delay. However, the ultimate goal of code dissemination optimization is to optimize the network performance. This performance includes network lifetime and code propagation delay. Therefore, this article aims directly at improving the overall performance of the network, not just reducing the number of transmissions. Based on this, this paper proposes a fast code propagation strategy from another novel point of view. The important difference between this strategy and previous strategies is that this strategy starts from the cross-layer optimization, increases the transmit power of the nodes in the energy-surplus region and reconstructs an optimized network to get better performance, which can reduce the number of broadcasts and significantly reduce the time required for code transmission. Therefore, the three important performance indicators of broadcast times, network lifetime, and transmission delay are better than with previous strategies. The magnitude of the improvement is difficult to determine in previous strategies. Specifically, the main innovations in this paper are as follows:

(1) An Adaption Broadcast Radius-based Code Dissemination (ABRCD) scheme is proposed to achieve lower code dissemination delays while retaining a higher network lifetime for WSNs. What is fundamentally different from previous strategies is that the strategy in this paper is to reduce the number of broadcasts and delay for code dissemination by adjusting the broadcast radius. At the same time, the network lifetime is not lower than in previous strategies. We note that the main task for WSNs is to monitor events and objects. Once a predefined event or physical phenomenon occurs, sensor nodes send the perceived data to the sink. Since the sink is the center of the entire network, the energy consumption of the nodes near to the sink is high, and the energy consumption of the nodes far from sink is low. Since the network lifetime depends on lifetime of the first dead node in the network, this paper proposes an ABRCD scheme that uses the same broadcast radius as the previous strategy in areas near to sink where the energy remain is tight, while the areas with energy surplus use a larger broadcast radius. In this paper, a theoretical analysis is given to determine the value of the broadcast radius in different areas of the network. This can make the code diffusion strategy with unequal broadcast radius get closer to energy consumption balance and improve the energy utilization ratio without affecting the network lifetime.
(2) An efficient and unequal-radius-based code dissemination algorithm is given in this paper for reducing transmissions and broadcast delay of code dissemination. The proposed code disseminating algorithm improves upon previous algorithms. The algorithm first constructs a broadcast backbone under an unequal broadcast radius scenario, and then broadcasts along the broadcast backbone. Since the broadcast radius of most areas in the ABRCD strategy is larger than that of previous strategy, the length of the constructed broadcast backbone path is shorter than the previous strategy and the number of nodes that can be transmitted to in a broadcast is also more than with the previous strategy, so the code diffusion algorithm proposed in this paper can effectively reduce the time and the number of transmissions required for code diffusion.
(3) Through our extensive theoretical analysis and simulation, we demonstrate that ABRCD scheme proposed in this paper has better performance. Compared to the previous schemes, our ABRCD scheme outperforms them in terms of all important performance indicators: (a) The number of transmissions can be effectively reduced. As confirmed by a large number of experiments, the transmissions of ABRCD are reduced compared with previous schemes by 36.18~94.27%; (b) The time for code dissemination is reduced by 41.11~78.42%; (c) The proposed strategy can effectively improve the energy efficiency by up to 583.42%. Finally, when all major performances are improved, its network life is higher than in previous strategies, which was difficult to achieve with those strategies.

The rest of the paper is organized as follows: Section 2 reviews related works compared with our scheme. Section 3 describes the network model and defines the problem statements of this paper. In Section 4, we give the ABRCD design scheme for WSNs. In Section 5, we do performance analysis on ABRCD. Section 6 presents experimental results and a comparison of the ABRCD scheme. We conclude this paper in Section 7.

## 2. Related Work

Sensor networks are being applied to all aspects of society, such as social networks [41,42,43,44], mobile networks [45], or forming the main body of the Internet of Things (IoT) [46,47,48,49], profoundly changing our ways of social interaction [42,47,48]. Energy is the most precious resource in wireless sensor networks [50,51]. To reduce energy consumption, it is necessary to minimize the number of broadcasts of nodes, thereby saving energy and improving the network lifetime. At the same time, as much data as possible needs to be transmitted to the destination. There are actually two different types of data transmission in wireless sensor networks. One is an *n*-to-1 data collection mode. In this mode, the sink is usually located in the center of the network, and *n* ordinary nodes are randomly deployed in the network. Nodes generate a data packet in one data collection cycle. Then, *n* data needs to be quickly transmitted to the sink. This is an *n*-to-1 data collection mode. The other data propagation model is studied in this paper. The program codes of the sink under this propagation mode need to be transmitted to all nodes in the network. This is a 1-to-*n* mode.

**Figure 1:**
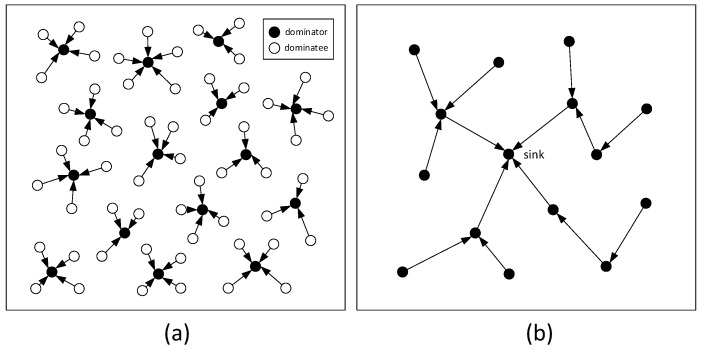
*n*-to-1 data collection method (**a**) Dominator collects data of its dominatees. (**b**) Dominator data reaches sink via multi-hop route.

The following describes the data propagation method in *n*-to-1 data propagation mode. One of the data collection methods called converged cast is a typical method for data collection, which can be seen in Figure 1 [52]. Each node in the network generates a data packet in one data collection period and needs to send it to the sink. Due to the redundancy between data, data fusion is used to collect data. Data fusion is an *n*-to-1 data fusion method. That is, after the *n* data packets meet, the merged data is still a data packet. To save energy and minimize delay, Figure 1 gives a concurrent data collection method [52]. First, the network is divided into multiple sets. There is only one dominator (black nodes in Figure 1) in each set. The other nodes are called dominatees. Any node in the network must belong to a set [52]. In this way, data in the entire network can be collected to sink through the following two processes. The first process is for each dominatee (white nodes in Figure 1) to send data to the dominator and for each dominator to fuse all the data received into a packet (see the left of Figure 1). Then, in the second step, dominators send data to the sink through a multi-hop route, as shown in the right of Figure 1 [52].

In this paper, we study a 1-to-*n* transmission method. That is, a source node transmits data to *n* nodes [25]. Such a method is often applied to diffuse program codes [25]. A sink disseminates the updated program codes to each node in the network, so that nodes can acquire new functions after updating the software [25]. Since the wireless network has the function of broadcasting, multiple recipients can receive data packets after one broadcast. Therefore, the use of broadcasting can substantially reduce the number of transmissions to nodes, thereby reducing the energy consumption of the network [25,53]. Researchers have summarized this problem as the issue of Minimum-transmission broadcast (MTB) [25]. One way to solve the MTB problem is to construct a Minimum Connected Dominating Set (MCDS) for the network [54]. Then the nodes on the MCDS broadcast to allow data to be transmitted to each node in the network.

Different from the node periodic awake/sleep mode of operation in this paper, the initial MTB study assumes that the nodes of the wireless network or the wireless ad hoc networks (WANETs) are all working continuously. Therefore, these kinds of studies often use a Set-Cover-based Approximation (SCA) scheme [28] (actually similar to MCDS). In the SCA scheme, there are two main steps: (a) Select a node set that can cover the entire network. These nodes are called covering nodes. This problem is the so-called set covering problem. (b) Construct a broadcast backbone based on these covering nodes. After the completion of the above, codes can be broadcasted along the broadcast backbone. Since nodes on the broadcast backbone cover all the nodes in the network, each node on the broadcast backbone broadcasts once, and it can ensure that codes are propagated to each node of the network. Therefore, constructing a backbone that can cover the entire network and reach the minimum transmissions becomes the key in this method. Different broadcast backbones require different numbers of broadcasts. Research in [28] shows that such an SCA scheme is a complete NP problem.

Then, some researchers proposed the MTB problem in the duty-cycle based network, called the MTB problem in duty-cycled networks (MTB-DC problem) [28]. In this study, nodes are working periodically. In general, one period is divided into *k* time slots. The node selects one of the time slots as a working slot and sleeps in other time slots. In such a network, the number of broadcasts required for code propagation will be much greater than the number when nodes are always working. This is because, for a covering node, when it broadcasts, the nodes in its coverage range are not all awake at the moment. Only nodes that are in the awake state of this time slot can receive broadcast packets. Therefore, in such a network, the most extreme situation is that if the nodes in the coverage node covering range choose *k* different time slots, respectively, the covering node needs to broadcast *k* times to transmit codes to each node. Thus, more broadcasts are generated than in the no duty-cycle based network. Obviously, the MTB scheme proposed in the previous no duty-cycle based network can be applied to the duty-cycle based network. Hong et al. [28] proposed a modified MTB scheme. In the modified MTB scheme, only a maximum of *k* broadcasts per node on the broadcast backbone are needed to ensure that each node can receive data. However, Hong et al. [28] confirmed that the performance of this modified strategy based on the original MTB scheme is not necessarily optimized. Therefore, Hong et al. [28] proposed two new MTB-DC schemes: the centralized SCA (CSCA) algorithm, and the distributed SCA (DSCA) algorithm, respectively. The complexity of these two algorithms is: for CSCA algorithm, 3(lnΔ+1) approximation ratio and $O\left(n^{3}\right)$ time complexity (Δ is the maximum degree of the network); for the DSCA algorithm, a constant approximation ratio and both linear time and message complexities.

In an MTB scheme, the purpose of reducing the number of broadcasts is to save energy, and reducing delay is another important research objective of this type of strategy. We proposed an adjustable duty cycle-based fast disseminate (ADCFD) scheme in [25] to minimize the number of broadcasts while reducing the time required for broadcasting for smart wireless software-defined networks. In the code transmission, the number and time of broadcasts required to broadcast are related to the duty cycle used by the node. In general, the greater the duty cycle of a node, the smaller the number of broadcasts and delay required for code transmission. The most specific example is that if the node is always awake without sleeping, i.e., the duty cycle is 1, then it is the same as the MTB in [54], and each covering node only needs to broadcast once, while the smaller the duty cycle, the larger the number of broadcasts and delay. For example, if a node only has one time slot awake in *k* time slots, it means that it needs to broadcast *k* times in the worst case. Obviously, the larger the number of *k* is, the larger the number of broadcasts is. Therefore, another effective way to reduce propagation times and delays is to increase duty cycle (decrease *k*), but increasing the duty cycle consumes more energy, thus reducing network lifetime. [25] found that during data collection, the area far from sink has energy surplus, so in the ADCFD scheme, the remaining energy of these regions is fully utilized to increase the duty cycle of nodes in this region so that the number of broadcasts and delay for nodes in these region can be decreased, and the performance of code transmission is improved.

There are some similar studies. For example, Wieselthier et al. [55] proposed a scheme, whose main idea is to build a minimum transmission tree to reduce transmission delay. Le Duc et al. [27] proposed a level-based approximation scheme. In his scheme, the nodes in the broadcast backbone remain active. Thus, data can be quickly transmitted to the broadcast backbone nodes far away from the data source, while other nodes not on the broadcast backbone work in the duty-cycled mode. In this way, as a whole, most nodes adopt a duty-cycled mode. Only the nodes on the broadcast backbone are always active, so the energy consumption is relatively small, but it can effectively reduce the delay. Similar research on duty cycle WSNs was also presented in [56,57].

## 3. System Model and Problem Statement

### 3.1. System Model

The software defined wireless sensor network (SDWSN) in this paper consists of m homogenous static sensor nodes $v_{i}|i\in \left\{1\ldots m\right\}$ and the sink $v_{0}$, so the node set can be indicated as $M≜\left\{v=v_{0},v_{1},v_{2},\;\ldots ,v_{m}\right\}$. The same as in previous research [27], the network can be abstracted as a unit disk graph *G* = (M, ℰ), and ℰ is the set of edges. A directed edge from node *u* to node *v* exists if Euclidean distance between *u* and *v* is within the broadcast radius of *u*. Broadcast radius of each node is variable, according [58]. The network radius is *R*. Sensor nodes are powered by battery with limited energy, while the energy of sink is unlimited.

**Figure 2:**
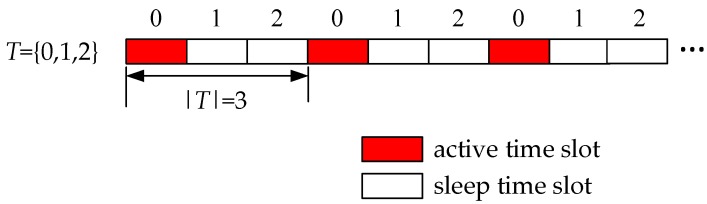
The example of active/sleep time slot.

During data transmission and reception, an uncoordinated duty-cycle mechanism is adopted, thus, it is necessary to conduct time synchronization among nodes. Global time is divided into several working periods. Each working period is composed of |*T*| time slots, where *T* is the set of time slots, indicated as *T* = {0,1,2, …, |*T*| − 1}. Each node *v* has two status, active and sleep, and chooses its active time slot $\tau_{v}$ randomly among *T*. A node is only able to receive in its active time slot but can wake up to transmit in any time slot. Considering the working period of a network is 3, then *T* = {0,1,2}. And a node choose its active time slot 0, then its active/sleep status is as shown in Figure 2.

The central node $v_{0}$ is responsible for disseminating codes to the entire network and collecting data of all sensors. The code dissemination we study in this paper can be described as: $v_{0}$ first collects information of network topology and active time slot of each node, then it builds the broadcast backbone and start to broadcast program codes to neighbor nodes, then the program codes are transmitted from those neighbor nodes to outside nodes, until program codes reach every node in the network.

### 3.2. The Energy Consumption Model

The energy consumption of a node contains two aspects: (1) The energy consumption for transmitting data packets $E_{i}{}^{T}$, (2) the energy consumption for receiving data packet $E_{i}{}^{R}$.

$E_{i}{}^{T}$ is described as follows:

(1) $$E_{i}{}^{T}=\{\begin{array}{c} Q_{i}{}^{T}E_{elec}+Q_{i}{}^{T}\varepsilon_{fs}r_{i}{}^{2},\;\;r_{i}<d_{0}\\ Q_{i}{}^{T}E_{elec}+Q_{i}{}^{T}\varepsilon_{amp}r_{i}{}^{4},\;\;r_{i}\geq d_{0} \end{array}$$

$Q_{i}{}^{T}$ is the amount of transmitting data bit of node $v_{i}$. $r_{i}$ is the broadcast radius of node $v_{i}$. $E_{elec}$ is the energy of transmitting circuit loss. $\varepsilon_{fs}$, $\varepsilon_{amp}$ is energy of power amplification loss for the Free Space Model and Multi-path Fading Model, respectively.

Energy consumption for data transmitting is a piecewise function. When the broadcast radius is less than threshold $d_{0}$, the Free Space Model is taken to calculate power amplification loss. When the broadcast radius is not less than $d_{0}$, power amplification turns to a Multi-path Fading Model.

$E_{i}{}^{R}$ is described as follows:

(2) $$E_{i}{}^{R}=Q_{i}{}^{R}E_{elec}$$

$Q_{i}{}^{R}$ is the amount of receiving data bit of node $v_{i}$. Thus, the total energy consumption for node $v_{i}$ is:

(3) $$E_{i}=\{\begin{array}{c} Q_{i}{}^{T}\left(E_{elec}+\varepsilon_{fs}r_{i}{}^{2}\right)+Q_{i}{}^{R}E_{elec},\;\;r_{i}<d_{0}\\ Q_{i}{}^{T}\left(E_{elec}+\varepsilon_{amp}r_{i}{}^{4}\right)+Q_{i}{}^{R}E_{elec},\;\;r_{i}\geq d_{0} \end{array}$$

The parameters adopted in the energy model are similar to those given in [57] and their values are listed in Table 1.

### 3.3. Problem Statement

This paper aims to design an ABRCD scheme. This scheme can reduce transmissions and broadcast delays, and at the same time, increase the network lifetime, energy utilization ratio compared with previous schemes. Thus, ABRCD aims to minimize transmissions, minimize delay, maximize lifetime and maximize energy utilization. Details of the four aspects are as follows:

(1) Minimization of transmissions. As a sensor has multiple neighbors and the chosen active time slots of neighbors are often different, a node often has to broadcast several times. Transmissions of the network is the sum of broadcast times of each node when program codes arrive at the edge of the network. LBAS provides a method to reach the minimum transmissions. The ABRCD scheme reduces transmissions further by enlarging the broadcast radius of some nodes. Thus, a node has more neighbors, bringing probability to reach more nodes in one broadcast. Let $B=\left(V_{B},E_{B}\right)$ denote the final backbone, where $V_{B}\in M$, and $E_{B}\in$ ℰ. Let ${ℑ}_{i}$ be the set of transmitting time slots of $v_{i}$, the value of transmissions can be calculated as:

(4) $$ℸ=\underset{v_{i}\in V_{B}}{\sum }\left|{ℑ}_{i}\right|$$

(2) Minimization of delay. The broadcast delay T is the number of time slots taken for codes to be transmitted from the code source to the boundary of a network. As the active time slots of neighbors are often different, there is a waiting time between two broadcasts. Suppose there are two neighbors *α*, *β* for a node, and broadcasting to *α* is ahead of *β*, the waiting time can be calculated as $\tau_{\beta }$ − $\tau_{\alpha }$ when $\tau_{\beta }$ ≥ $\tau_{\alpha }$, or $\tau_{\beta }$ − $\tau_{\alpha }$ + |*T*| when $\tau_{\beta }$ < $\tau_{\alpha }$. It is known that decrement in the number of transmissions leads to decrement in broadcast delay. Thus, ABRCD provides a reduction in broadcast delay by generating less number of transmissions. Let $\varrho_{v}$ be the parent of node *v* in the backbone. The delay taken for codes to arrive at node *v* can be calculated as:

(5) $$T_{v}=\{\begin{array}{c} T_{\varrho_{v}}+\tau_{v}-\tau_{\varrho_{v}},\;\;\tau_{v}\geq \tau_{\varrho_{v}}\\ T_{\varrho_{v}}+\tau_{v}-\tau_{\varrho_{v}}+\left|T\right|,\tau_{v}<\tau_{\varrho_{v}} \end{array}$$

and the delay for program codes to arrive at the network edge can be calculated as:

(6) $$T=\max T_{v}$$

(3) Maximization of network lifetime. Lifetime in this paper is defined as the number of periods the network works until the first dead node appears, for after the first node dies, the topology of network is destroyed. The connectivity and coverage of the network are severely affected, leading the network to be unable to play the designated function. ABRCD generates an improvement in network lifetime because enlarging the broadcast radius for nodes near an original hot-spot makes them reach the sink directly without forwarding by nodes in the hot-spot, and not only in hot-spot, but also nodes in other areas forward less due to the larger broadcast radius. Thus, the highest data burden due to forwarding is reduced and the highest energy consumption (energy consumption of nodes in hot-spot area) is reduced. Lifetime can be expressed as:

(7) $$P=\frac{E_{0}}{\max_{i\in \left\{1\ldots m\right\}}E_{i}}$$

(4) Maximization of energy utilization. In ABRCD, network energy consumption is mainly determined by the data load and broadcast radius. The energy consumption theory in [57] is that energy consumption is much more in hot-spot areas than that at areas far from the sink. The network topology becomes broken and the entire network can’t be used when the first dead node appears. At this time about 90% energy is not used. The original idea for ABRCD is to enlarge the radius for nodes far from the sink to better use the energy surplus at those nodes, since radius enlarging brings less data burden, as the number of forwarding times is less. The energy consumption of some nodes is reduced. However, as lifetime is improved, even though energy consumption of some nodes in one period is less, with more working periods and larger radius of nodes far from sink, energy utilization is improved. Energy utilization ratio can be calculated as:

(8) $$\psi =\frac{\sum_{1\leq i\leq m}PE_{i}}{\sum_{1\leq i\leq m}E_{0}}$$

Summing up, the optimization goal of ABRCD in this paper is:

(9) $$\{\begin{array}{c} \min\left(ℸ\right)=\min(\underset{i\in B}{\sum }\left|{ℑ}_{i}\right|)\\ \;\;\;\min\left(T\right)=\min\max T_{v}\\ \max\left(P\right)=\max\frac{E_{0}}{\max_{i\in \left\{1\ldots m\right\}}E_{i}}\\ \max\left(\psi \right)=\max\frac{\sum_{1\leq i\leq m}PE_{i}}{\sum_{1\leq i\leq m}E_{0}} \end{array}$$

## 4. The Design of the ABRCD Scheme

### 4.1. Research Motivation of ABRCD

**Figure 3:**
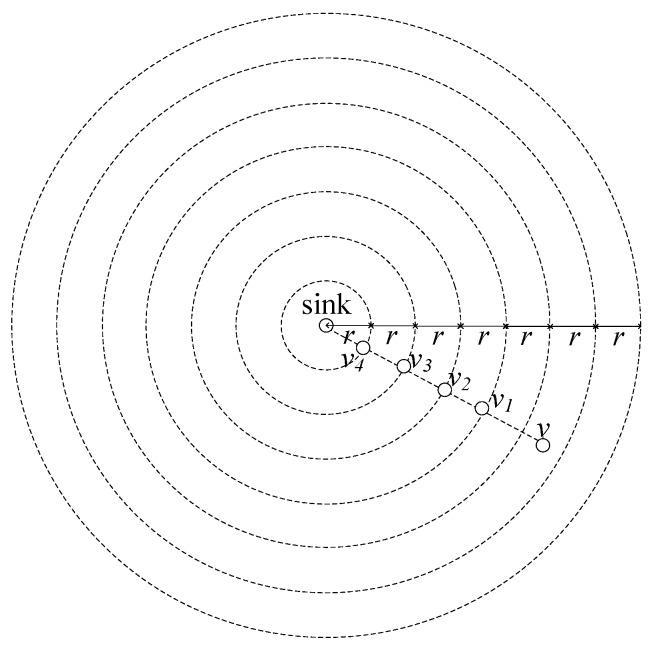
Zoning by the same *r*.

During data collection, all sensors send their sensed data to a sink. Nodes near the sink carry a great amount of data, causing much energy consumption of these nodes. Once the energy runs out in a hot-spot area, the topology of the entire network is destroyed. The network is not able to work appropriately, while there is a lot of energy surplus in nodes far from sink. In order to make use of the energy surplus in nodes far from the sink, we enlarge the transmission radius of these nodes, and is different from previous studies in which the node transmission radius are all same as in Figure 3. Thus, delay can be reduced while lifetime is not affected.

**Figure 4:**
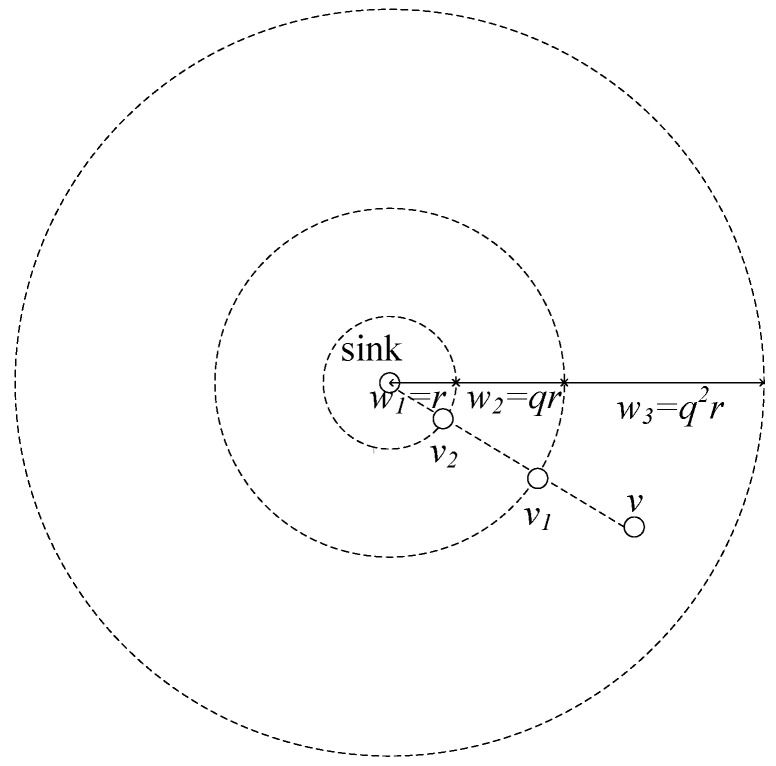
Zoning in geometric progression.

LBAS provides an algorithm that aims to reach the minimum transmissions [27]. On the basis of LBAS, ABRCD aims to generate less number of transmissions further by enlarging the broadcast radius of nodes far from the sink. The method to assign a radius to each node is to first zone the network into different areas, and then assign a corresponding broadcast radius to the nodes in each area. Our idea is to zone networks according to their distance to the sink. We zone the entire network into several rings, whose width $w_{i}$ grows by geometric progression as shown in Figure 4**.** Nodes in a certain zone have a corresponding radius, which has the same length as the width $w_{i}$. If the original radius is *r*, and zoning distance set can be denoted as $\left\{w_{1},w_{2},\;\ldots ,w_{N}\right\}$. $d_{1}$ = *r*, common ratio *q* can be defined as:

$$q=\frac{w_{i+1}}{w_{i}},i\in \left\{1\ldots N\right\}$$

If *q* = 2, and *R* = 7*r*, network zoning is shown in Figure 4**,** while the original zoning is shown in Figure 3.

**Definition** **1 (Layer).** *Given node v, at $d_{v}$ away from sink, layer of v is defined as the hop count from sink to v under ideal conditions. The ideal conditions are that there is always a forwarding node on the intersection of the edge of zoning area and the straight line from sink to the node v. Like $v_{4}$, $v_{3}$, $v_{2}$, $v_{1}$ as forwarders for v in Figure 3, and $v_{2}$, $v_{1}$ as forwarders for v in Figure 4.*

**Definition** **2 (Level).** *Given node v, at $d_{v}$ away from sink, level of v is defined as hop count from sink to v under practical condition. The practical condition is the hop count based on the actual topology.*

**Theorem** **1.** *In LBAS, the number of layers of the entire network can be calculated as:*

$$L=\lceil \frac{R}{r}\rceil$$

*Layer of v can be calculated as:*

$$L_{v}=\lceil \frac{d_{v}}{r}\rceil$$

*In ABRCD, the number of layers of the entire network can be calculated as:*

(10) $$N=\lceil \log_{q}\left(1+\frac{R}{r}\left(q-1\right)\right)\rceil$$

*Layer of v can be calculated as:*

(11) $$n_{v}=\lceil \log_{q}\left(1+\frac{d_{v}}{r}\left(q-1\right)\right)\rceil$$

**Proof.**  In LBAS, *R* ≤ *r* × *L*, $d_{v}$ ≤ *r* × $L_{v}$, where *L* and $L_{v}$ are both integers making the right side of equations just not less than the left side. Thus:

$$L=\lceil \frac{R}{r}\rceil ,\;L_{v}\left\lceil \frac{d_{v}}{r}\right\rceil$$

Like in Figure 3, *L* = 7, $L_{v}$ = 6.

In ABRCD, *R* and $d_{v}$ are sum of some width growing in geometric progression, and *R* ≤ *r* × $\frac{1-q^{N}}{1-q}$, $d_{v}$ ≤ *r* × $\frac{1-q^{n_{v}}}{1-q}$, where *N* and $n_{v}$ are both integers making the right side of equation just bigger than the left side. Thus:

$$N=\lceil \log_{q}\left(1+\frac{R}{r}\left(q-1\right)\right)\rceil$$

$$n_{v}=\lceil \log_{q}\left(1+\frac{d_{v}}{r}\left(q-1\right)\right)\rceil$$

Like in Figure 4, *N* = 3, $n_{v}$ = 3. □

**Theorem** **2.** In ABRCD, the broadcast radius for each node can be calculated as:

(12) $$r_{v}=w_{n_{v}}=rq^{n_{v}-1}$$

**Proof.**  As the radius-assigning method introduced before, nodes in a certain zone are assigned a radius that has the same length as the width of the zone. The width of the zone is related to the layer of the node, and the layer of node *v* can be calculated using Equation (11). Then the broadcast radius of node *v* can be calculated using general term formula for geometric sequence:

$$r_{v}=w_{n_{v}}=rq^{n_{v}-1}$$

Like in Figure 4, $n_{v}$ = 3, $r_{v}$ = 4*r*. □

**Figure 5:**
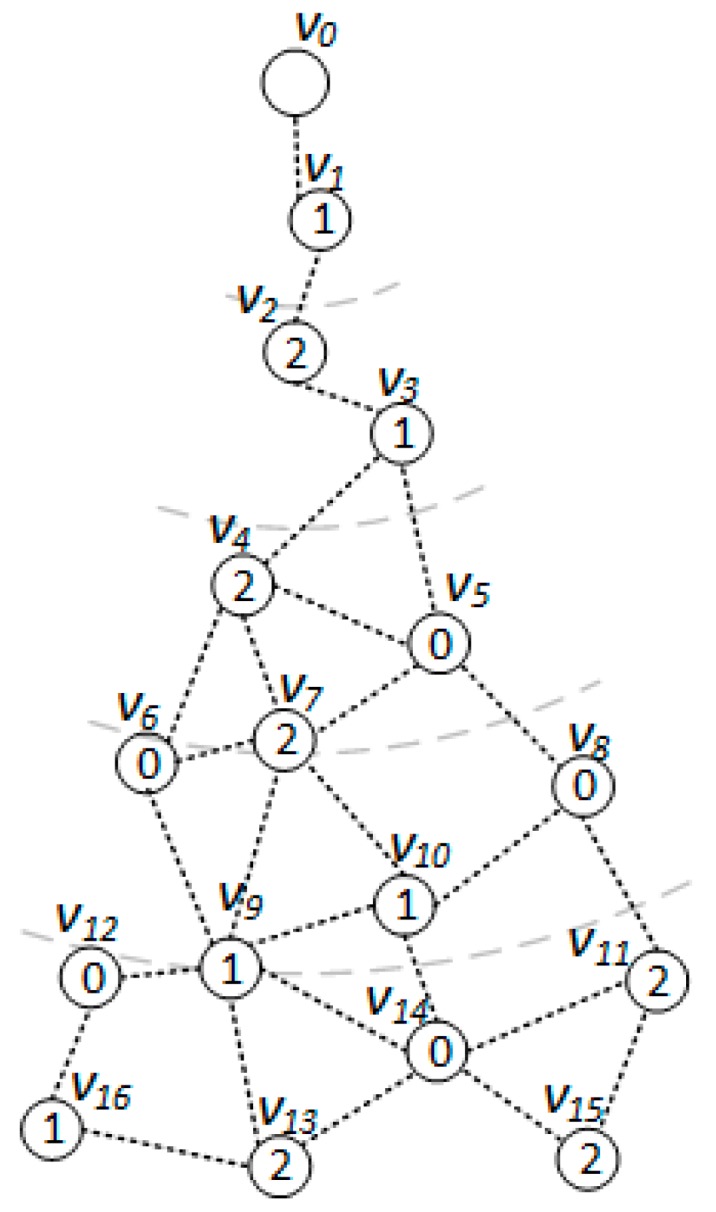
Original physical link.

**Figure 6:**
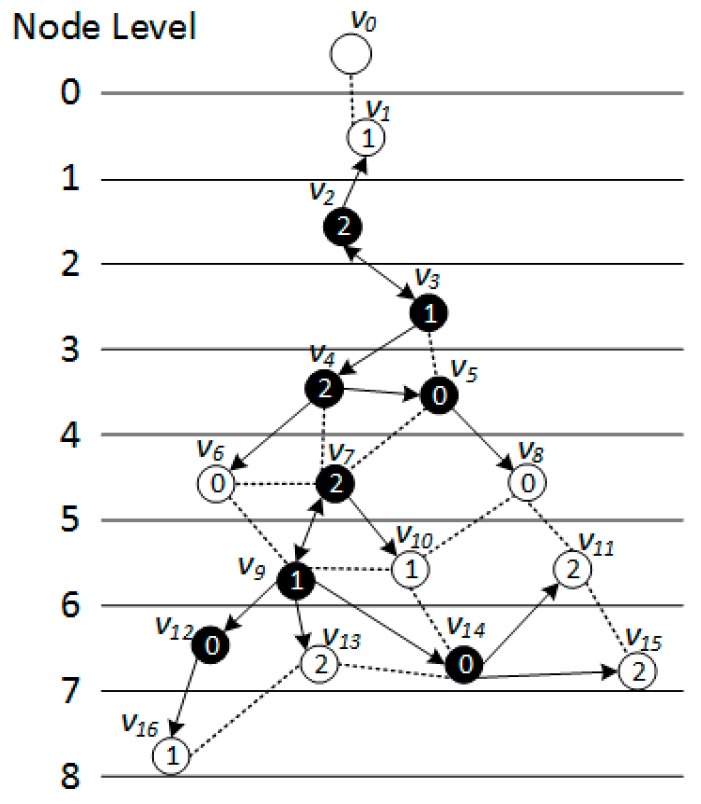
Level-clear figure with minimum for LBAS covering node set.

**Figure 7:**
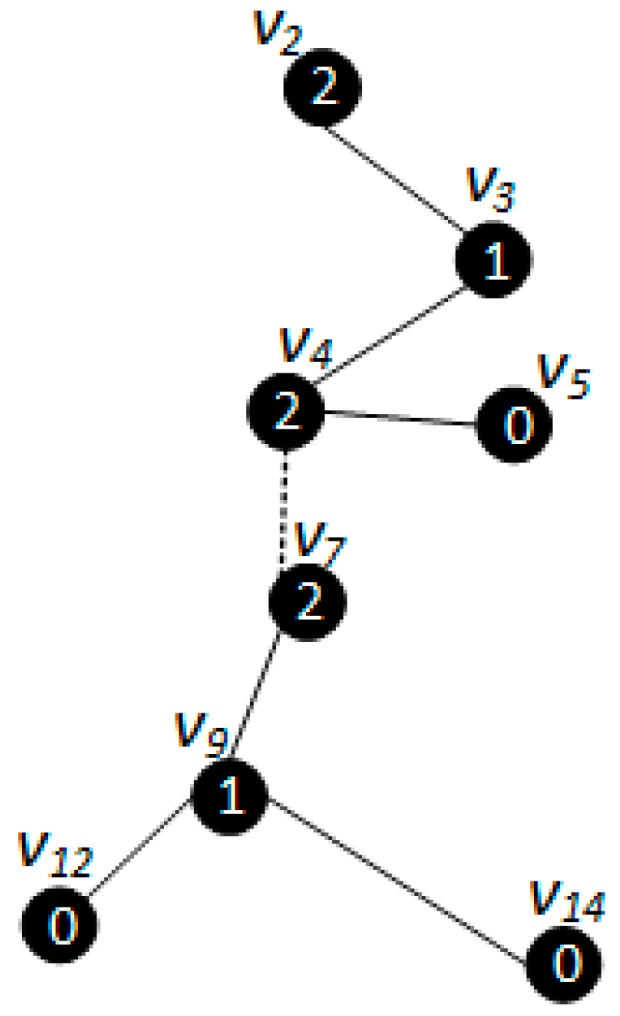
Broadcast backbone for LBAS.

**Figure 8:**
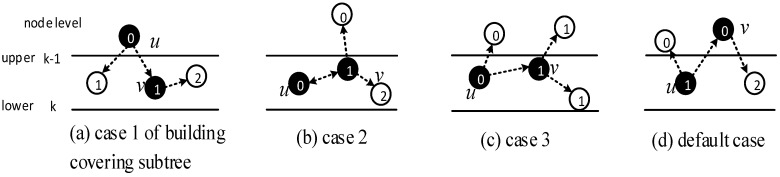
Building covering sub-tree.

**Figure 9:**
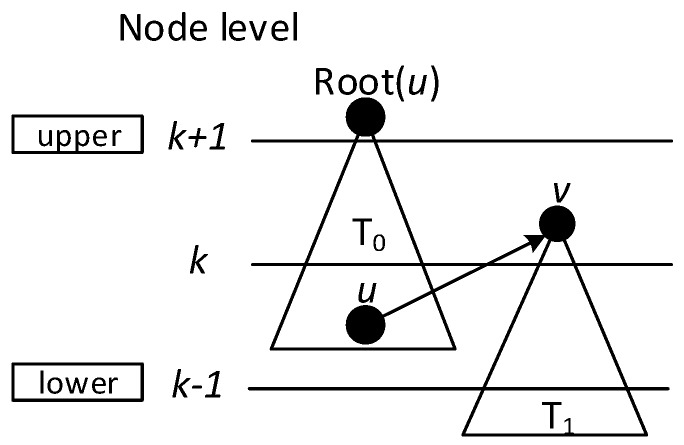
Case 1 in finalizing backbone.

**Figure 10:**
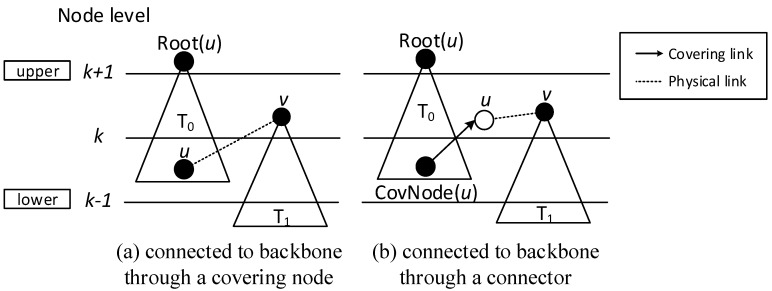
Case 2 in finalizing backbone.

**Figure 11:**
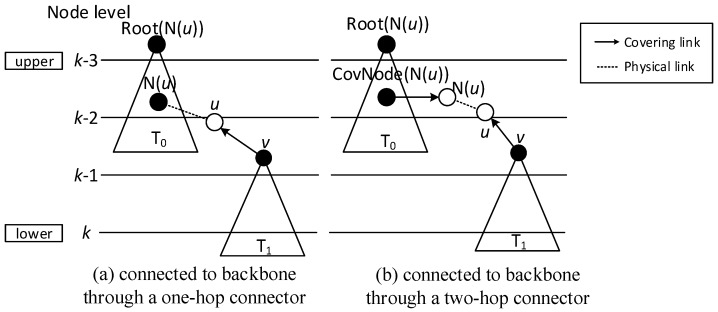
Case 3 in finalizing backbone.

**Figure 12:**
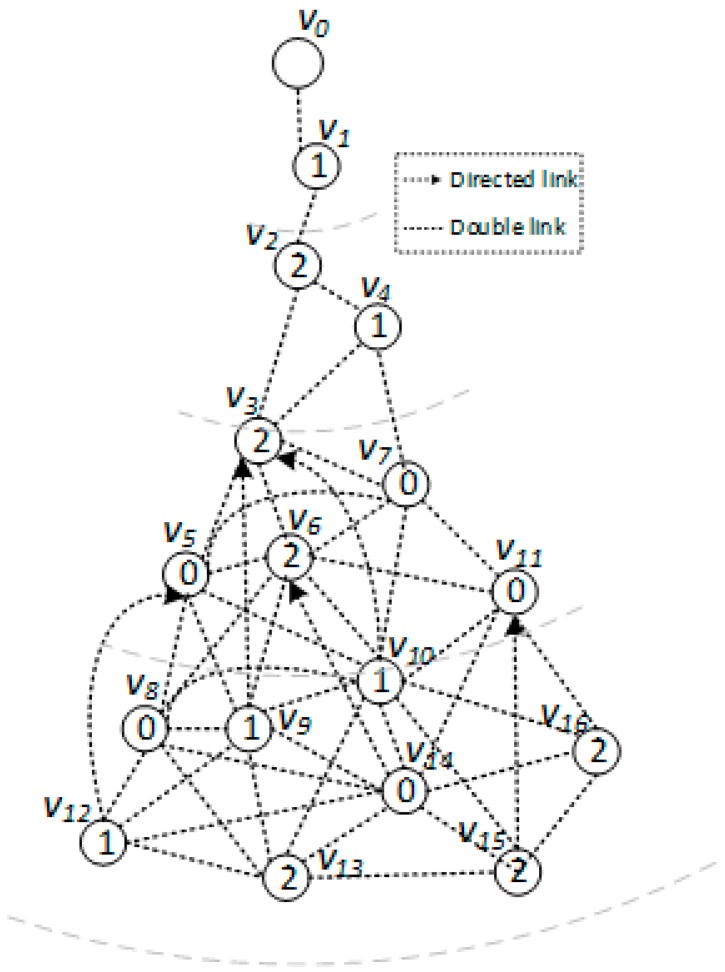
Original physical link.

**Figure 13:**
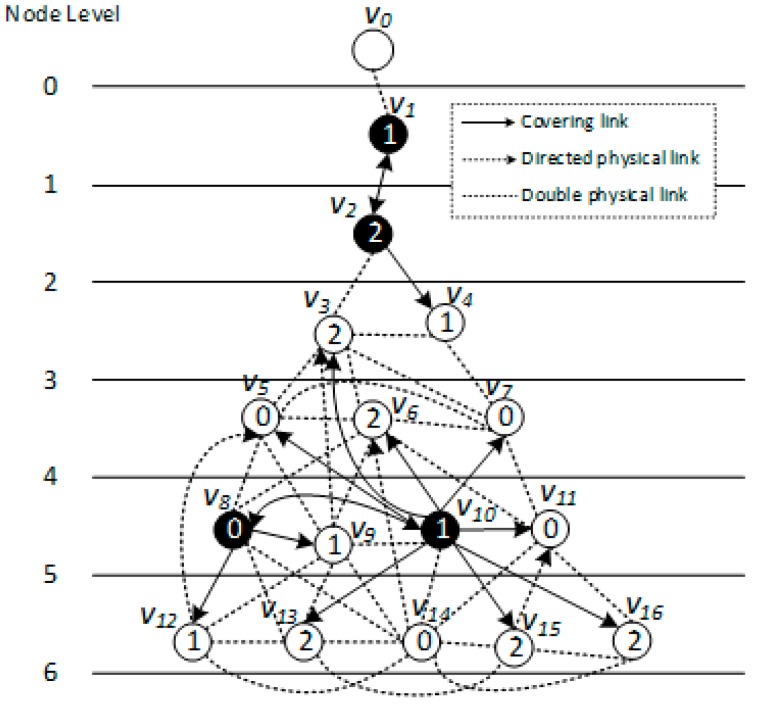
Minimum covering node set for ABRCD.

**Figure 14:**
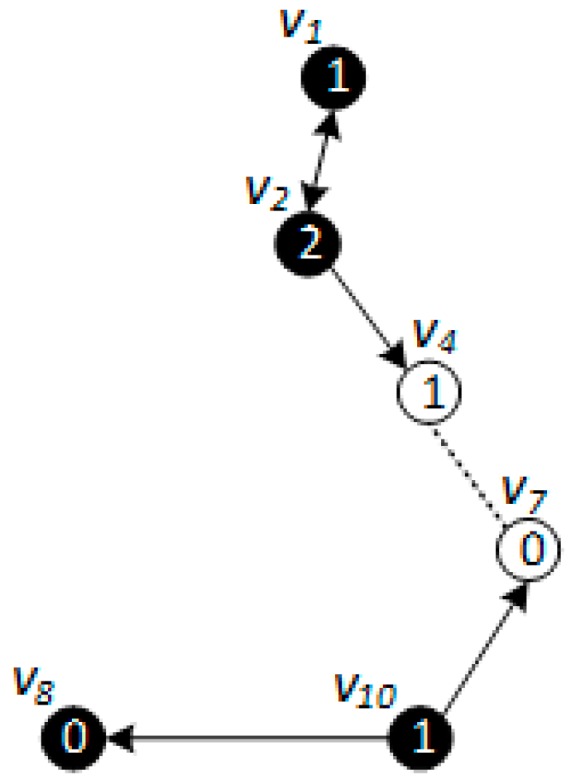
Broadcast backbone for ABRCD.

**Figure 15:**
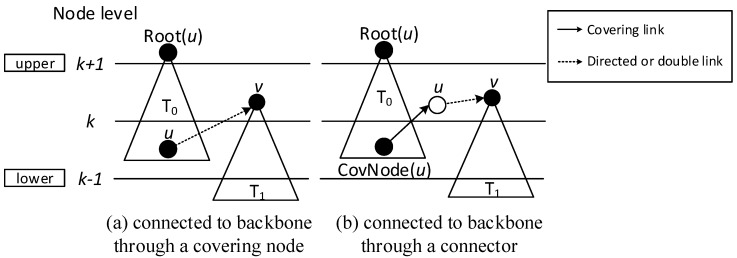
Connecting ${\;T}_{1}$ to $T_{0}$ using one-hop connector.

**Figure 16:**
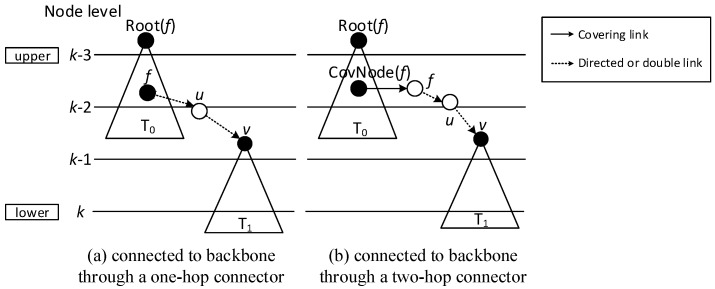
Connecting ${\;T}_{1}$ to $T_{0}$ using one-hop and two-hop connectors.

**Example** **1.** *Figure 5, Figure 6 and Figure 7 show the backbone construction process adopting the LBAS scheme, and Figure 8, Figure 9, Figure 10 and Figure 11 are the algorithm illustration of LBAS schemes. Figure 12, Figure 13 and Figure 14 show the backbone construction process adopting the ABRCD scheme with q = 1.1, and Figure 15 and Figure 16 are the algorithm illustration of LBAS schemes. The node distributions of the two schemes are the same, and the number of time slots |T| = 3. An original network topology with every node assigned the same radius r is shown in Figure 5. The network has five layers. When adopting the ABRCD scheme, the topology of network becomes that in Figure 12. The network has four layers this time. The following describes the process of adopting LBAS (Figure 5, Figure 6 and Figure 7) first and then the process of ABRCD (Figure 12, Figure 13 and Figure 14). Descriptions below change ${ℑ}_{i}$ and $\varrho_{v}$ in Section 3 to ST(i) and Pr(v) for the convenience of algorithm descriptions.*

LBAS provides an algorithm to construct a broadcast backbone for reducing broadcast delay and transmission times. Before program codes start to be broadcasted, the broadcast backbone should be built. LBAS is composed of two parts: find the minimum covering node set and building the broadcast backbone. Building the backbone involves two stages. The first is to connect covering nodes from upper to lower level, building covering sub-trees. The second is to connect the sub-trees from lower to upper level, finishing the backbone. Thus, the broadcast backbone construction is accomplished by three steps: (1) Find the minimum covering node set. (2) Build the covering sub-tree. (3) Finalize backbone.

(1) Find the minimum covering node set

For each time slot, a covering set is composed of a minimum number of nodes which can cover all nodes active at that time slot. Nodes in such a set are called covering nodes, and nodes covered by these covering nodes are called covered nodes. A greedy algorithm is adopted to find the minimum covering set. For every time slot *i:*

① Find a node *v* covering most uncovered nodes in slot *i* as covering node and mark nodes covered already. Add *i* to covering time slots of *v*. That is, ST(*v*) = ST(*v*)∪{*i*}.
② Find other covering nodes until all nodes in time slot *i* are all covered.

Nodes find above make up the covering node set for time slot *i*. Covering node sets for all time slots(0~|T| − 1) can be found in the same way. And each covered node *v* knows its covering node, identified as CovNode(*v*).

Figure 6 is a level-clear graph of Figure 5**.** The minimum covering node set is also shown in Figure 6, where the minimum covering set for time slot 0 is {$v_{4}$, $v_{5}$, $v_{9}$}, for time slot 1 is {$v_{2}$, $v_{7}$, $v_{12}$}, for time slot 2 is {$v_{3}$, $v_{9}$, $v_{14}$}.

(2) Build the covering sub-tree

When building the sub-tree, there are four cases:

- Case 1: If a covering node *v* is covered by another one *u* at upper level, like in Figure 8a, we assign *u* to be the parent of *v*. That is, Pr(*v*) = *u*. And Root(*v*) = Root(*u*). In Figure 6, $v_{3}$ is covered by $v_{2}$, so Pr($v_{3}$) is $v_{2}.$ Root($v_{3}$) is $v_{2}$, because $v_{2}$ is the root itself.
- Case 2: If two covering nodes, *u* and *v*, cover each other and they are at the same level, like in Figure 8b, then the one with more neighbors is selected to be the parent of the other. If they have the same number of neighbors, then use id to break the tie. Suppose *u* is the parent of *v*. If Root(*u*) is not set yet, *u* itself is selected as the root. And Root(*v*) = Root(*u*). In subsequent Figure 13, $v_{8}$ and $v_{10}$ covers each other, while $v_{10}$ has more neighbors, so Pr($v_{8}$) is $v_{10}$ and $v_{10}$ itself is a root, so Root($v_{8}$) is set as $v_{10}$.
- Case 3: If two covering nodes, *u* and *v* are at the same level and *u* covers *v*, like in Figure 8c, then Pr(*v*) = *u* and no cycle shall be generated. If Root(*u*) is not set yet, *u* itself is selected as the root. And Root(*v*) = Root(*u*). In Figure 6, $v_{4}$ covers $v_{5}$, so Pr($v_{5}$) is $v_{4}$. Root($v_{5}$) is set as Root($v_{4}$), That is, Root($v_{5}$) = $v_{2}$.
- Default Case: If a covering node v doesn’t trigger any of the above cases and it just forms a covering sub-tree with a single node itself, like in Figure 8d, it is the default case. Then v is selected to be the root. Like in Figure 6, when $v_{2}$ and $v_{7}$ first added to build the sub-tree, they are added itself, with no parent.

(3) Finalizing backbone

When finalizing the backbone, from lower to upper level, for the covering nodes which don’t have parent, there are three cases:

- Case 1: Node *v* can find a covering node *u* that covers it at lower level, but Root(*u*) is at upper level, like in Figure 9, then Pr(*v*) = *u*. Root(*v*) is updated as Root(*u*). Our example doesn’t trigger this case, but it is obvious that the first stage only connects the lower covering nodes to the upper ones or covering nodes at the same level. There could be a covering sub-tree whose root node *v* is at the upper level compared with its covering node *u*, but at the lower level compared with Root(*u*).
- Case 2: If node *v* can’t find a parent in Case 1, then it tries to find a neighbor *u* as parent. *u* must satisfy one of the following two conditions: (1) *u* is a covering node in the sub-trees and Root(*u*) is at upper level than *v*. Like in Figure 10a, then Pr(*v*) = *u*. (2) *u* is a connector, and Root(CovNode(*u*)) is at upper level than *v*, like in Figure 10b, then *u* is added to the backbone, and Pr(*v*) = *u*, Pr(*u*) = CovNode(*u*), Root(*v*) = Root(CovNode(*u*)). For all the above two cases, the active time slot of *v* is added to covering time slots of *u*. That is, ST(*u*) = ST(*u*)∪{AT(*v*)}. In Figure 7, Pr($v_{7}$) is $v_{4}$, because $v_{4}$ is a node satisfying condition (1).
- Case 3: If node *v* can’t find a parent in all two above cases, then it tries to find a covered node *u* as parent. *u* must satisfy one of the following two conditions: (1) *u* is a connector, and *u* has a neighbor N(*u*) already in the backbone, whose root is at upper level than that of *v*. Like in Figure 11a, then *u* is added to the backbone and Pr(*u*) = N(*u*), Pr(*v*) = *u*, Root(*v*) = Root(N(*u*)). (2) *u* is a connector, and *u* can find a neighbor N(*u*) as another connector, and CovNode(N(*u*)) is at upper level than that of *v*. Like in Figure 11b, then *u* and N(*u*) are added to the backbone and Pr(N(*u*)) = CovNode(N(*u*)), Pr(*u*) = N(*u*), Pr(*v*) = *u*. For all the above two cases, the active time slot of *v* is added to covering time slots of *u*. That is, ST(*u*) = ST(*u*)∪{AT(*v*)}. And ST(N(*u*)) = ST(N(*u*))∪{AT(*u*)}.

In addition, we use the order of Breadth-first Search (BFS) starting from code source as id to nodes, because in this way breaking ties by id can lead to a broadcast tree with the lowest depth, making it more probable to diminish delays. The process of data dissemination from sink to sensor nodes is shown in Table 2, where information of nodes receiving data in every time slot is concluded. The number of transmissions of adopting LBAS scheme is 9 and delay is 15.

The ABRCD scheme first assigns a broadcast radius according to Equation (12). The topology of adopting ABRCD is shown in Figure 12. It can be observed that directed links exist in ABRCD, because different nodes may have different radii. Situations can arise where node *v* can cover node *u*, while node *u* can’t cover node *v*. Minimum covering set finding and covering sub-tree building are the same as in LBAS, just with changed transmitting radii in different areas (Figure 12). As the two schemes adopting the same algorithm in the above two stages, we take situations in Figure 13 to better explain LBAS in a foregoing passage.

While finalizing backbones has slight differences because of the existence of directed links, the little modification needed in Case 2 and Case 3 is that connectors need to be nodes that can cover node *v*, instead of just node *v* covering the connector. The modified Case 2 and Case 3 are illustrated in Figure 15 and Figure 16 respectively. It can be observed that:

1. “Physical link” in Case 2 is replaced by “Directed or double link”, because it should be guaranteed that codes can be transmitted from node *u* to node *v*. “Physical link” and the “Covering link” of *v* in Case 3 is replaced by “Directed or double link” for the same reason.
2. “N(*u*)” is replaced by “*f*” in Case 3, because when adopting ABRCD, *u* is required to be the neighbor of *f*, so that program codes can be transmitted from *f* to *u*.

The modification can be observed in Figure 13. When connecting covering node $v_{10}$ to upper covering node $v_{2}$, $v_{3}$ owns smaller id number than $v_{7}$, it is selected as the connector with a higher priority in Case 3. $v_{3}$ should have been chosen as connector if adopting LBAS scheme, yet it isn’t the connector in ABRCD, because it is unable to transmit data to $v_{10}$, So, instead $v_{7}$ is chosen as the connector.

When there are directed edges, the above algorithm is very effective and guarantees data can be transmitted to sub-trees at lower level in simulations. Details of the modification are given in Section 4.2.

The final backbone of ABRCD is shown in Figure 14, and the process of data dissemination from sink to sensor nodes is shown in Table 3. The number of transmissions in ABRCD is 5 and delay is 11. Compared with LBAS in Table 2, the number of transmissions is reduced by 44.4% and delay by 26.7%.

From Figure 5 and Figure 12, it can be observed that in ABRCD, nodes far from the sink cover more neighbors with larger radius, which makes it probable for them to cover more nodes in one transmission. Thus, the number of transmissions decreases, and delay also becomes smaller at the same time.

### 4.2. Algorithm of ABRCD

As mentioned in Section 4.1, modifications for the LBAS backbone finalizing part are needed because of the existence of directed links. Case 1 doesn’t need to be changed, because a covering node *u* for *v* can satisfy codes transmitted from *u* to *v*, while Case 2 and Case 3 should be modified as follows:

- Case 2: If node *v* can’t find a parent in Case 1, then it tries to find a node *u* that can cover it. That is, *v*∈N(*u*). *u* must satisfy one of the following two conditions: (a) *u* is a covering node in the sub-tree and Root(*u*) is at upper level than *v*. Then Pr(*v*) = *u*. (b) *u* is a connector, and Root(CovNode(*u*)) is at upper level than *v*. Then *u* is added to the backbone, and Pr(*v*) = *u*, Pr(*u*) = CovNode(*u*), Root(*v*) = Root(CovNode(*u*)). For all the above two cases, the active time slot of *v* is added to covering time slots of *u*. That is, ST(*u*) = ST(*u*)∪{AT(*v*)}.
- Case 3: If node *v* can’t find a parent in all two above cases, then it tries to find two forwarders *u* and *f*. *v*∈N(*u*), *u*∈N(*f*). *u* and *f* must satisfy one of the following two conditions: (a) *u* is a connector, *f* is a covering node already in the backbone, and Root(*f*) is at upper level than *v*. Then Pr(*u*) = *f*, Pr(*v*) = *u*, Root(*v*) = Root(*f*). (b) *u* is a connector, *f* is another connector, and Root(CovNode(*f*)) is at upper level than *v*. Then Pr(N(*u*)) = CovNode(N(*u*)), Pr(*u*) = *f*, Pr(*v*) = *u*. For all the above two cases, the active time slot of *v* is added to covering time slots of *u*. That is, ST(*u*) = ST(*u*)∪{AT(*v*)}. And the active time slot of *u* is added to covering time slots of *f*, ST(*f*) = ST(*f*)∪{AT(*u*)}.

Thus, the pseudo code of ABRCD is shown in Algorithm 1.

**Algorithm 1:** The ABRCD scheme**Input:** A set of nodes, $M≜\left\{v=v_{0},v_{1},v_{2},\;\ldots ,v_{m}\right\}$, with their coordinates. And AT(*v*), ∀*v*∊M 1.   **for** each node $v_{i}$ from $v_{1}$ to $v_{m}$
**do** 2.        Calculate $d_{v_{i}}$ using its coordinates 3.        Calculate $n_{v_{i}}$ using Equation (11) 4.        Calculate $r_{v_{i}}$ using Equation (12) 5.   **end for** 6.   Construct graph *G* = ( M, ℰ) based on coordinates and radius of each node 7.   Conduct BFS on *G* starting from $v_{0}$, obtain the level of each node L(*v*), and take the order of BFS as id for each node 8.   Find the Minimum Covering Node Sets $C_{i}$, *i*∊*T* 9.   Build Covering Sub-tree, and obtain the set of root and parent of each node, Root and Pr 10.   Finalize Backbone: //Backbone is denoted as B 11.   **for**
*l* in [max({L(*x*)}) .. 1] **do** 12.        **for** each node *v*, L(*v*) = *l*
**do** 13.             **if** L(Root(CovNode(*v*))) < L(*v*) **then**      //Case 1 14.                  Pr(*v*) ← CovNode(*v*) 15.             **else** 16.                  Find a forwarder*u*, satisfying       //Case 2 17.                  (1) *v*∈N(*u*) 18.                  (2) [*u*∈B and L(Root(*u*)) < L(*v*)] or [L(Root(CovNode(*u*))) < L(*v*)] 19.                  **if** such a forwarder *u* exists **then**
20.                       Pr(*v*) ← *u*
21.                       AddToBackbone(*u*, CovNode(*u*), AT(*v*)) 22.                  **else** //Case 3 23.                       Find two forwarders *u* and *f*, satisfying 24.                       (1) *v*∈N(*u*) and *u*∈N(*f*) 25.                       (2) [*f*∈B and L(Root(*f*)) < L(*v*)] or [L(Root(CovNode(*f*))) < L(*v*)] 26.                       Pr(*v*) ← *u*
27.                       Pr(*u*) ← *f*
28.                       AddToBackbone (*f*, CovNode(*f*), AT(*u*)) 29.                       AddToBackbone (*u*, *f*, AT(*v*)) 30.                  **end if**
31.             **end if**
32.             Root(*v*) ← Root(Pr(*v*)) 33.        **end for** 34.   **end for** 35.   **procedure**AddToBackbone(*x*, *p*, *t*) 36.   **if**
*x*∊B **then**
37.        B ← B∪{*x*} 38.        Pr(*x*) ← *p*
39.        Root(*x*) ← Root(*p*) 40.   **end if** 41.   ST(*x*) ← ST(*x*)∪{*t*} 42.   **end procedure** **Output:** B, ST and Pr

## 5. Theoretical Analysis

### 5.1. Analysis of Energy Consumption

Data load and energy calculation adopt method in [57], but as the radius for every node is not the same, there is no continuous mathematical formula that can be provided to do the calculation, so we provide a discrete method (Algorithm 2) to measure the data load and energy consumption for nodes at a certain distance away from sink.

**Algorithm 2:** Discrete method to calculate data load and energy consumption**Input:** The whole network radius *R*, original broadcast radius *r*, common ratio *q*, the probability of generating data λ and energy parameters in Table 1 1.   Initializetotal_*Q* to all zeros/*total_$Q_{i}$ denotes a set of total data load in the Sector *i* */ 2.   Initialize*Q* to all zeros/*$Q_{i}$ is a set of average data load for each node in Sector *i* */ 3.   **for**
*i* in [*R*...1] **do** 4.   /*i is the distance from current node *v* to sink*/ 5.        Treat *i* as $d_{v}$ and calculate $n_{v}$ using Equation (11) 6.        Calculate $r_{v}$ using Equation (12) 7.        total_*Q*(*i*) = total_*Q*(*i*) + *i*
8.        tmpi = *i* − ⌊$r_{v}$⌋ 9.        **if** tmpi > 0 **then**
10.             total_*Q*(tmpi) = total_*Q*(tmpi) + total_*Q*(*i*) 11.        **end**
12.        *Q*(*i*) = (total_*Q*(*i*)/*i*)λ
13.        Calculate energy consumption *E*(*i*) using Equation (3) 14.   **end for** 15.   **Output:**
*Q* and *E*

**Figure 17:**
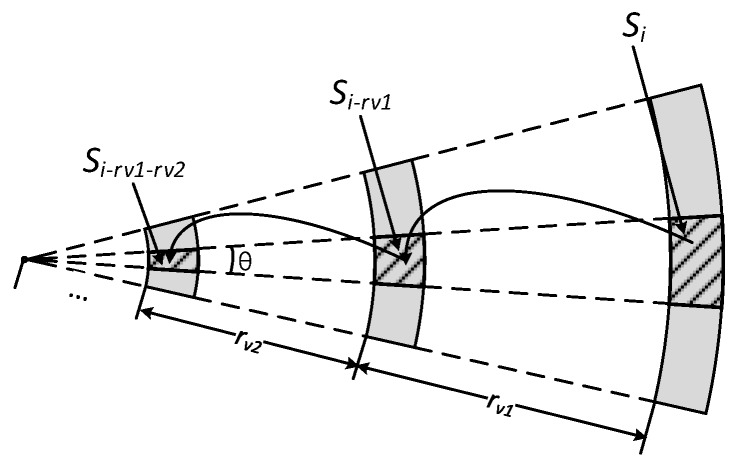
Process of data collection.

Algorithm 2 first initializes the total data load set total_$Q_{i}$ and the average data load set $Q_{i}$ for each sector to all zeros. Here Sector *i* denotes a small sector area where nodes are *i* m away from sink. In Figure 17, $S_{i}$ denotes Sector *i*. In [57], each sector will forward data towards sink to sector *r* away from itself. While in ABRCD, broadcast radius is variable, and each sector will forward data towards sink to sector $r_{v}$ away from itself. As the width b for every sector is very small, the area of each sector can be calculated by:

$$A_{i}=\theta ib$$

Suppose the density of nodes is *ρ*, then data generated in $S_{i}$ is θibρλ. When nodes in $S_{i}$ send data to sector $r_{v}$ away from itself, the average data burden for each node in the latter is:

$$Q_{i-r_{v}}=\theta ib\rho \lambda /(\theta \left(i-r_{v}\right)b\rho =\frac{i}{i-r_{v}}\lambda$$

It can be observed that the average data load is only related to the distance from the current node to the sink, and the distance can represent the data load generated in the current node area. We define total_*Q* to record the total data load at each distance. Line 7 is to add data generated to the total data load in the current area. Data generated in the current area doesn’t have to be received but must be transmitted. Line 9~11 is to measure total data load at $r_{v}$ away from current node after data transmitting to it. Then line 12 calculates the average data load for each node in current area.

Figure 17 shows the process of data collection. Line 7 achieves the calculation of data generated in $S_{i}$, $S_{i-r_{v1}}$, $S_{i-r_{v1}-r_{v2}}$, … Line 10 achieves the process of data transmission from $S_{i}\;\mathrm{to}\;S_{i-r_{v1}}$ and $S_{i-r_{v1}}$ to $S_{i-r_{v1}-r_{v2}}$ … Line 12 achieves to calculate average data on each node for $S_{i}$, $S_{i-r_{v1}}$, $S_{i-r_{v1}-r_{v2}}$, …

$Q_{i}$ is the set denoting the amount of transmitting data at certain distance. $Q_{i}$ − λ denotes the amount of receiving data. Then energy consumption is calculated by Equation (3).

Analysis is performed with R = 400, r = 40, λ = 0.1, and with *q* = 1.5, *q* = 2.0, *q* = 2.5, *q* = 3, *q* = 3.2 respectively. *E*(*i*) in Algorithm 2 is taken as energy consumption measurement.

**Figure 18:**
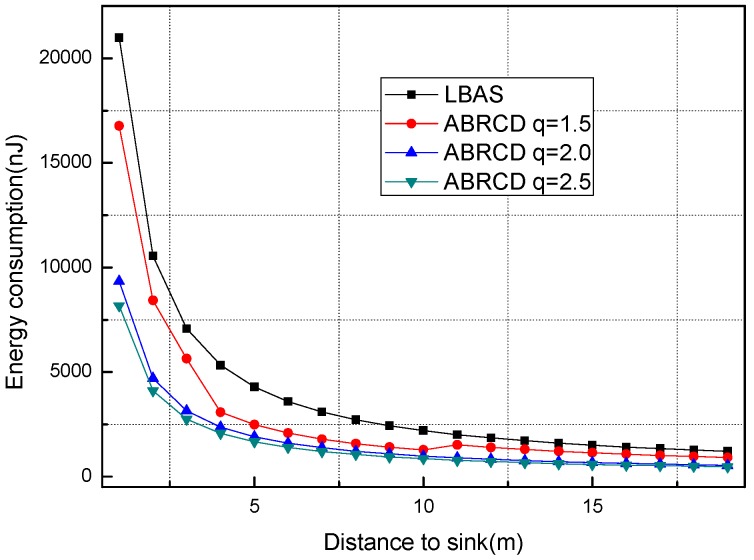
Energy consumption of nodes 1~19 m away from sink with different *q* (1.5, 2.0 and 2.5).

**Figure 19:**
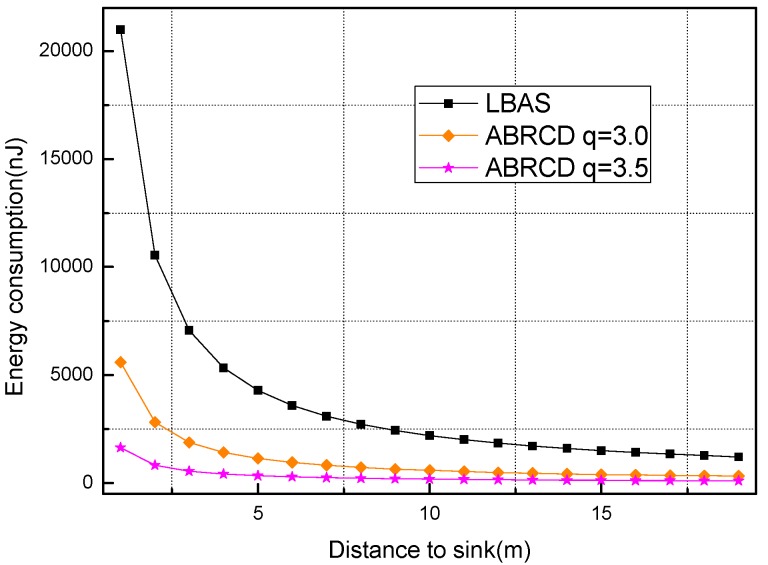
Energy consumption of nodes 1~19 m away from sink with different *q* (3.0, 3.5).

**Figure 20:**
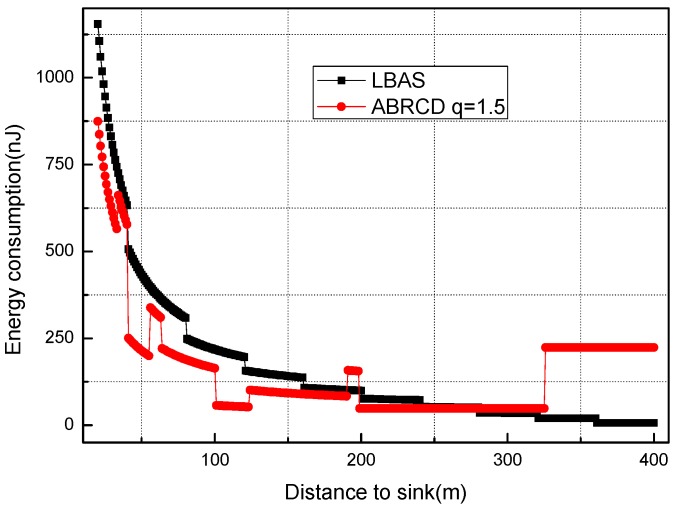
Energy consumption of nodes 20~400 m away from sink with *q* = 1.5.

**Figure 21:**
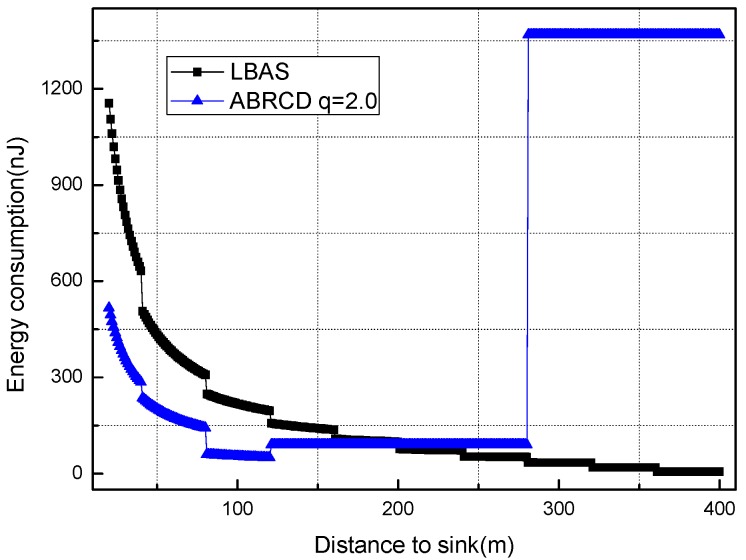
Energy consumption of nodes 20~400 m away from sink with *q* = 2.0.

**Figure 22:**
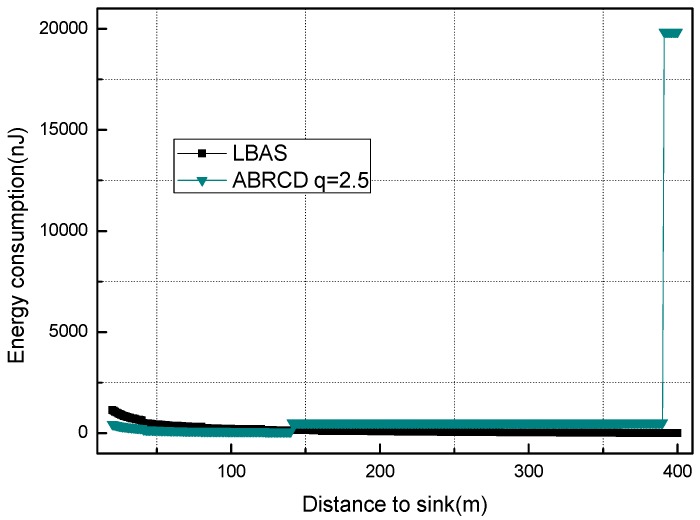
Energy consumption of nodes 20~400 m away from sink with *q* = 2.5.

**Figure 23:**
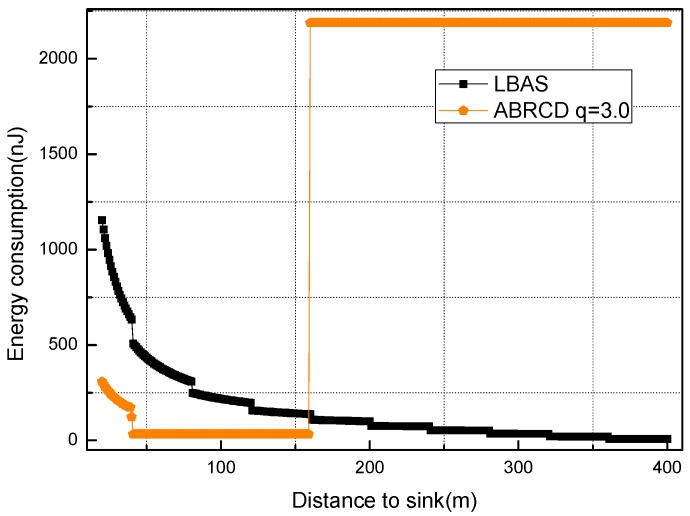
Energy consumption of nodes 20~400 m away from sink with *q* = 3.0.

**Figure 24:**
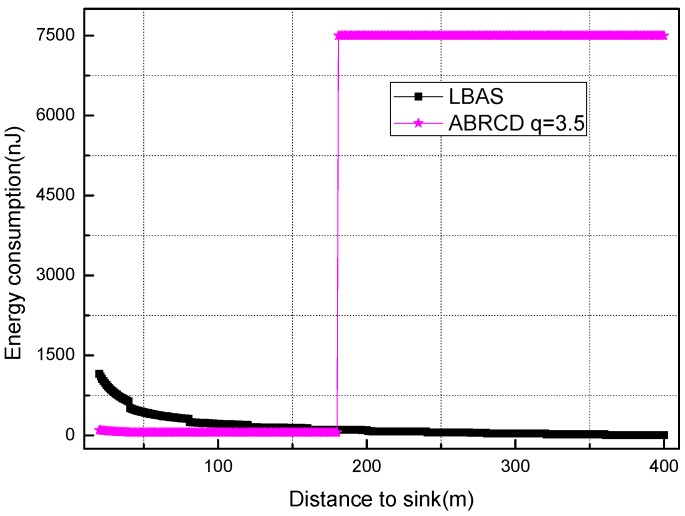
Energy consumption of nodes 20~400 m away from sink with *q* = 3.5.

Figure 18, Figure 19, Figure 20, Figure 21, Figure 22, Figure 23 and Figure 24 shows the energy consumption of LBAS and that of ABRCD with a selected common ratio *q*. The curve of ABRCD is not as smooth as that of LBAS, because calculation of the data load for ABRCD is achieved by a discrete method, while in LBAS, the radius of each node is the same, so a continuous mathematical formula can be used. When using a discrete method, the node receiving data is not exactly $r_{v}$ away from current node, but the lower bound of $r_{v}$ away. Thus, some nodes have to forward additionally while some forward less.

At areas near to sink (1~19 m), Figure 18 and Figure 19 show that energy consumption in ABRCD is not bigger than that in LBAS with any selected *q*. Energy consumption in original hot-spot area decreases because the data load decreases. The data load decrease in hot-spot areas is caused by more nodes reaching the sink immediately with the larger broadcast radius. Thus, nodes in original hot-spot areas don’t have to forward as much data as before. Somehow, the hot-spot area is widened.

At areas far from sink (20~400 m), Figure 20, Figure 21, Figure 22, Figure 23 and Figure 24 show that energy consumption of ABRCD is very high at areas far from sink, while lower than that of LBAS at some nearby areas. That shows that the large radius at far areas reduces the data burden at nearby areas. For *q* = 1.5, the energy consumption at far area is raised to about 250 nJ and for *q* = 2.0, about 1350 nJ. However, Figure 22 and Figure 24 (see along with Figure 18 and Figure 19) show that *q* = 2.5 and *q* = 3.5 are not proper to choose for conduction, because they generate so large a radius at far areas that the energy consumption at the far areas is even more than that at the hot-spot. When *q* = 2.5, energy consumption at far areas is almost 20,000 nJ, while approximately 5000 nJ at the hot-spot. When *q* = 3.5, energy consumption at far area is almost 7500 nJ, while approximately 2500 nJ at the hot-spot. This is not conducive to energy balancing and prolonging the network lifetime.

During several trials and analysis, we find that energy consumption at far areas overwhelms that at near area when *q* is not less than 3.2, and *q* between 3.0 and 3.2 achieve nearly the same effect. Thus, *q* = 3.0 is the most energy-balanced common ratio.

### 5.2. Analysis of Energyutilization Ratio and Network Lifetime

**Figure 25:**
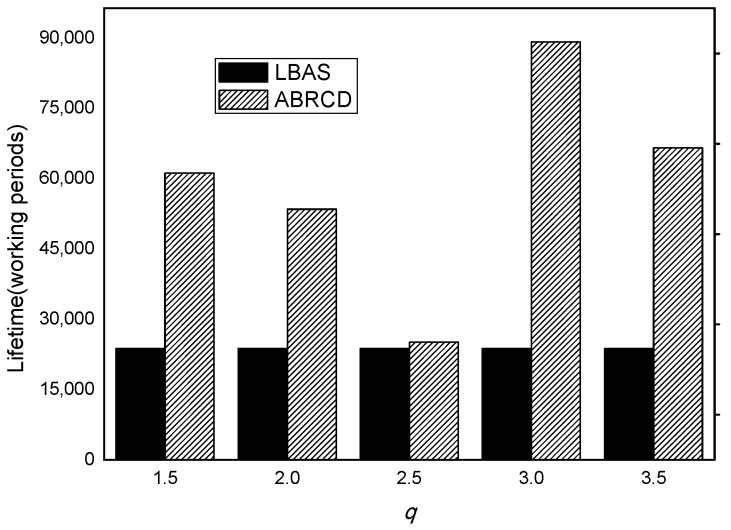
Lifetime with different *q.*

The lifetime of LBAS and ABRCD is calculated as Equation (7). As is shown in Figure 25, ABRCD outperforms LBAS in terms of lifetime, because of the reduction of the highest energy consumption. Among the chosen common ratios, *q* = 3.0 brings the best performance, increasing the lifetime of the network up by 274.99%. This is due to the advantage of energy balancing. Other common ratios prolong the lifetime by 157.20%, 124.92%, 5.79%, 179.92% with *q* = 1.5, *q* = 2.0, *q* = 2.5, *q* = 3.5, respectively.

**Figure 26:**
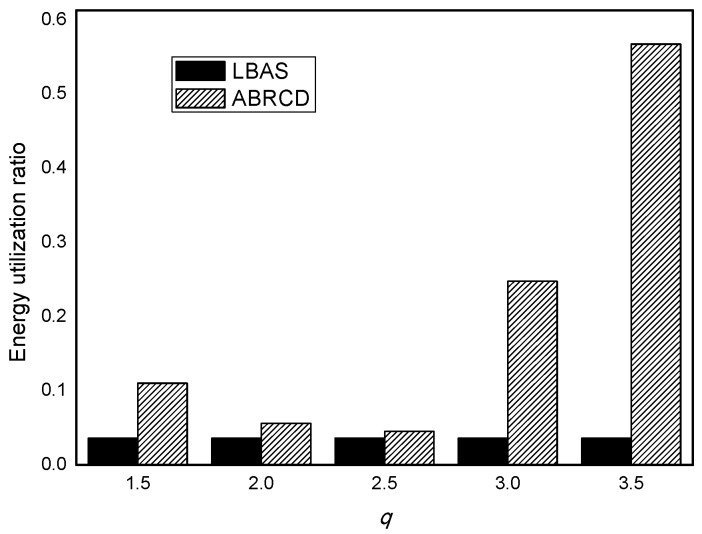
Energy utilization ratio with different *q.*

The energy utilization ratio of LBAS and ABRCD is calculated as Equation (8). In Figure 26, ABRCD outperforms LBAS in terms of energy utilization ratio. The effective energy utilization of ABRCD is mainly because of lifetime improvement and radius enlarging. ABRCD improves the energy utilization ratio by 204.70%, 54.14%, 25.14%, 583.42%, 1466.30% with *q* = 1.5, *q* = 2.0, *q* = 2.5, *q* = 3.0, *q* = 3.5, respectively.

## 6. Experimental Results Analysis of the ABRCD Scheme

### 6.1. Transmissions Analysis

Simulations are conduction of Algorithm 1 using Matlab, which is an h-language for algorithm development, data visualization, data analysis and numerical calculation. Constants adopted in conduction are as follows: radius of the whole network *R* = 400 m, the original transmitting radius r = 40, packet generation possibility is λ = 0.1. Other parameters setting refers to Table 1. Moreover, three proper value of common ratio *q* 1.5, 2.0, 3.0 mentioned before are applied in the simulation to see their impact.

**Figure 27:**
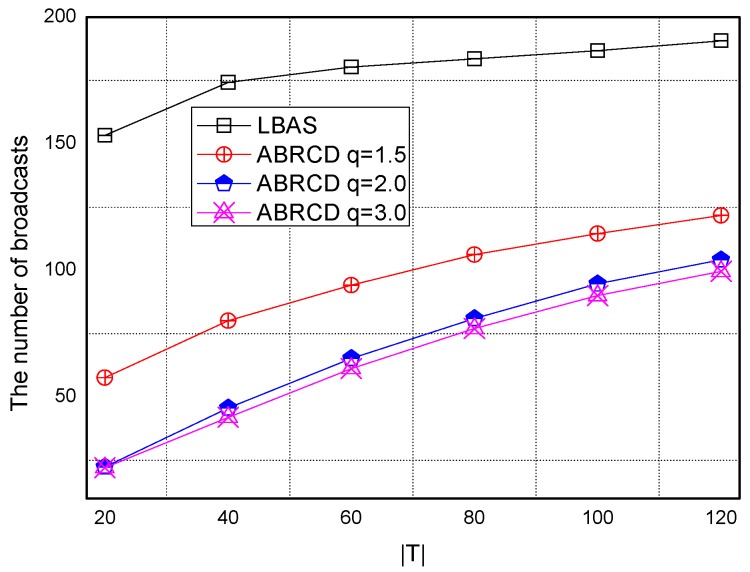
The impact of |*T*| and *q* with fixed network size = 200 on broadcasts.

**Figure 28:**
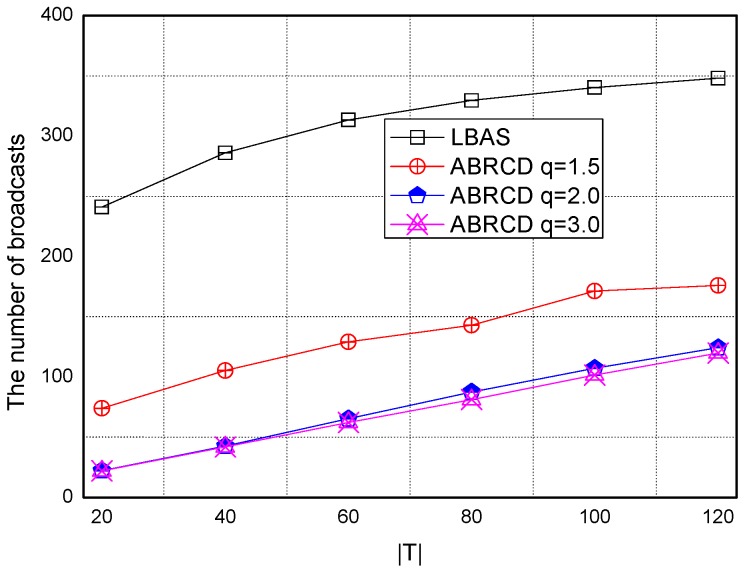
The impact of |*T*| and *q* with fixed network size = 400 on broadcasts.

**Figure 29:**
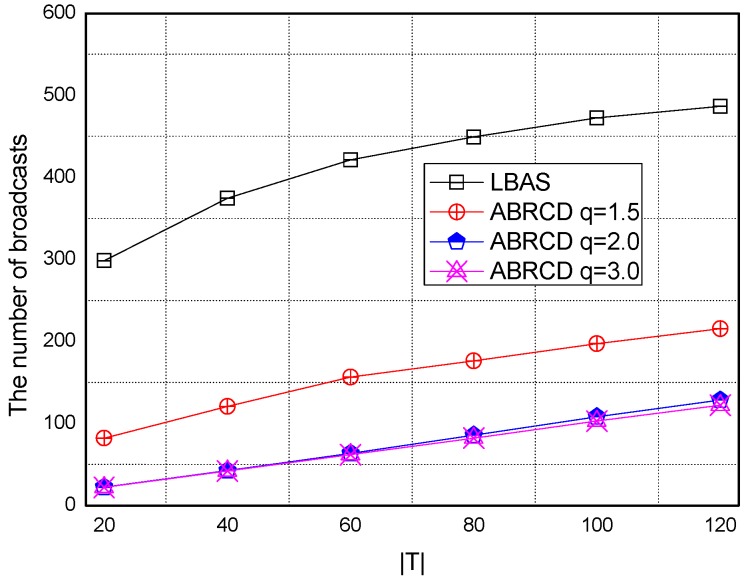
The impact of |*T*| with fixed network size = 600 on the number of broadcasts.

**Figure 30:**
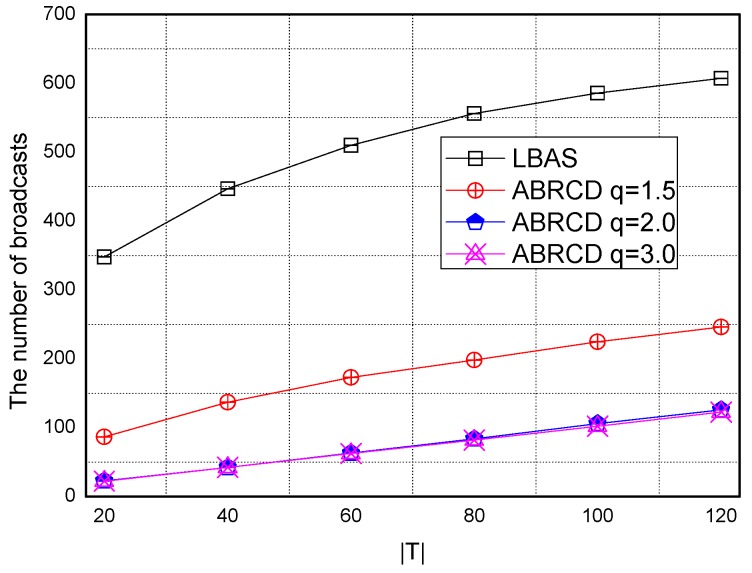
The impact of |*T*| with fixed network size = 800 on the number of broadcasts.

**Figure 31:**
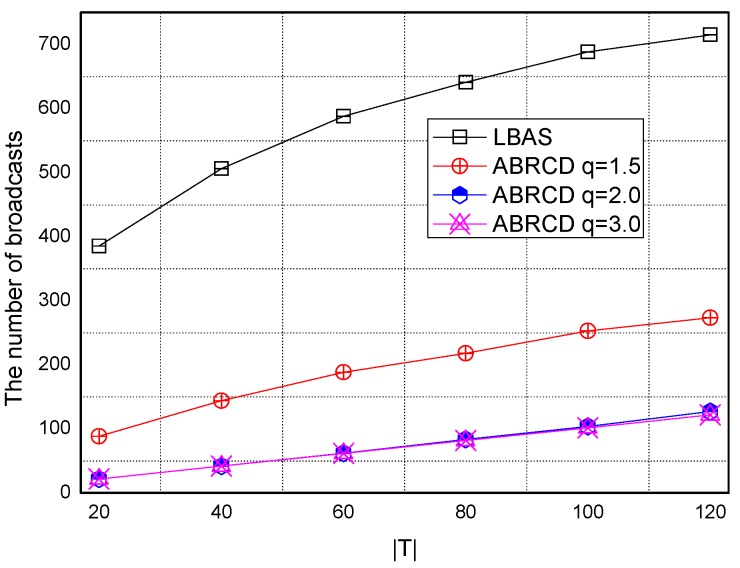
The impact of |*T*| with fixed network size = 1000 on the number of broadcasts.

Transmissions of ABRCD is calculated as Equation (4). B and ${ℑ}_{i}$ is from the output of Algorithm 1. The impact of total number of time slots |*T|* is shown in Figure 27, Figure 28, Figure 29, Figure 30 and Figure 31, with 200, 400, 600, 800, 1000 fixed nodes, respectively.

It can be observed that ABRCD generates less transmissions than LBAS, and larger *q* performs better. The number of broadcasts ABRCD generates is reduced up to 36.18~94.27% compared with LBAS. It is because ABRCD assigns large transmitting radius to nodes far from the sink. These nodes have more neighbors, thus, there is more probability to reach more covered nodes in one transmission. A larger radius generates fewer hop counts for codes to disseminate data from the code source to the edge of the network. Thus, the number of broadcasts is less than that of LBAS. Moreover, the ratio of reduction of ABRCD over LBAS decreases with higher |*T*|. This is because with higher |*T*|, the number of neighbors receiving codes during one transmission is reduced. When every neighbor has different active time slot with each other, the advantage of codes reaching more nodes in ABRCD is totally lost.

Both schemes generate a larger number of broadcasts when |*T*| is higher. This is obvious because increasing the number of time slots causes a covering node to need more covering time slots to transmit. When every neighbor has different active time slots with each other, the advantage of covering node set reaching the most covered nodes in one time slot is totally lost.

**Figure 32:**
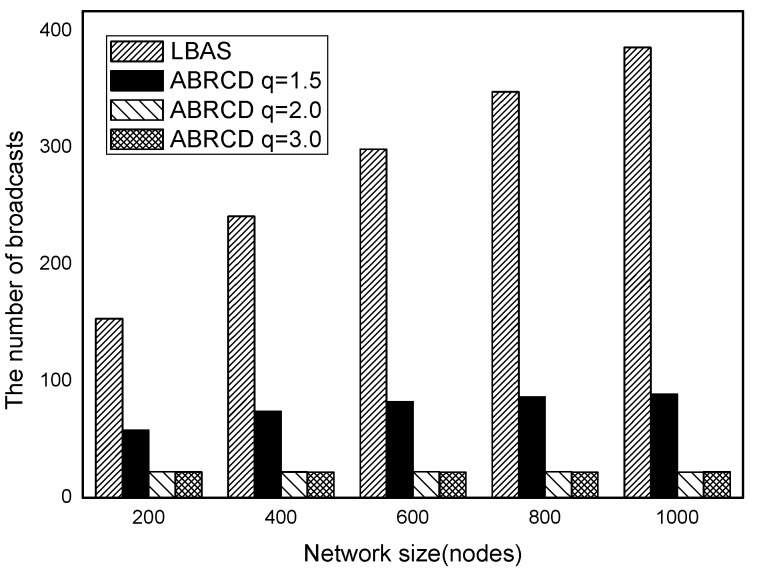
The impact of network size with fixed |*T*| = 20 on the number of broadcasts.

**Figure 33:**
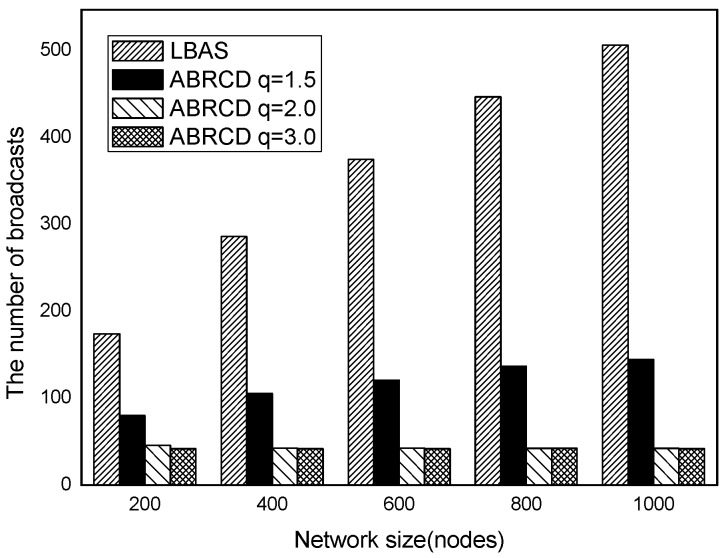
The impact of network size with fixed |*T*| = 40 on the number of broadcasts.

**Figure 34:**
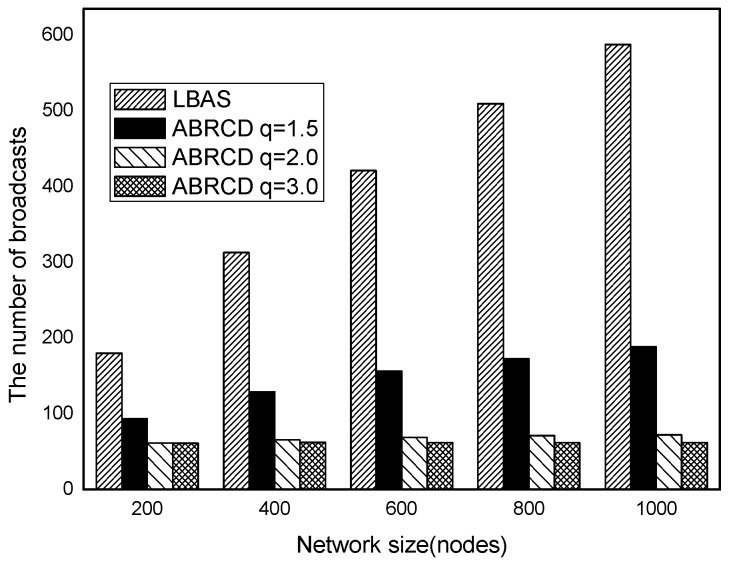
The impact of network size with fixed |*T*| = 60 on the number of broadcasts.

**Figure 35:**
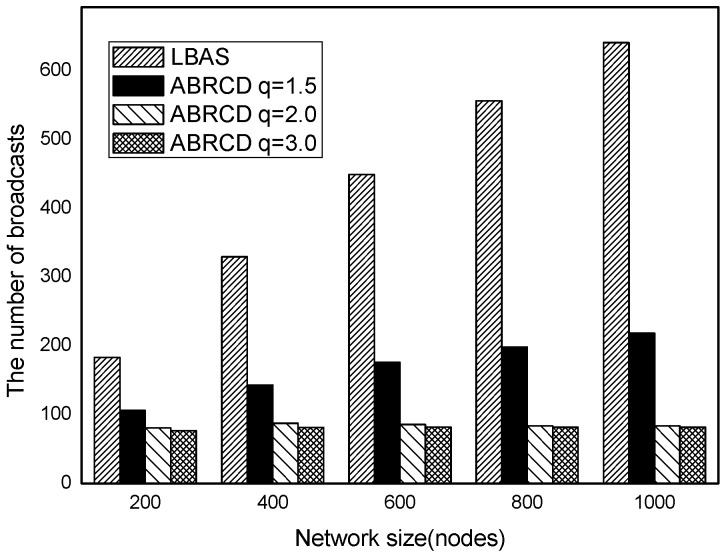
The impact of network size with fixed |*T*| = 80 on the number of broadcasts.

**Figure 36:**
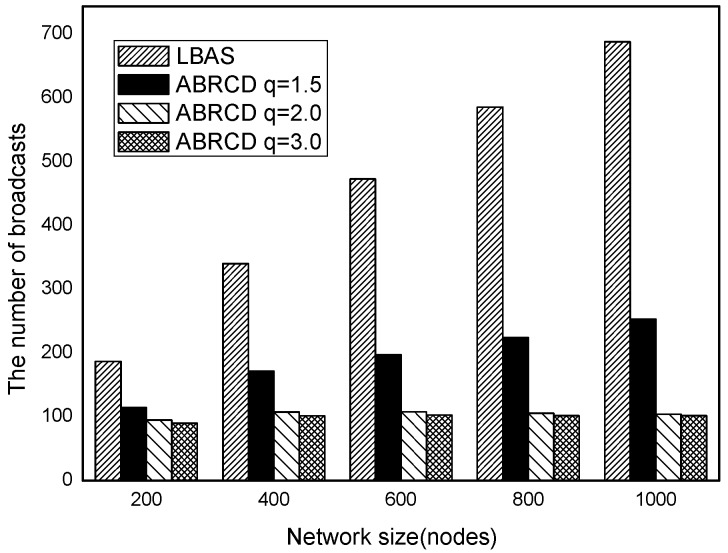
The impact of network size with fixed |*T*| = 100 on the number of broadcasts.

**Figure 37:**
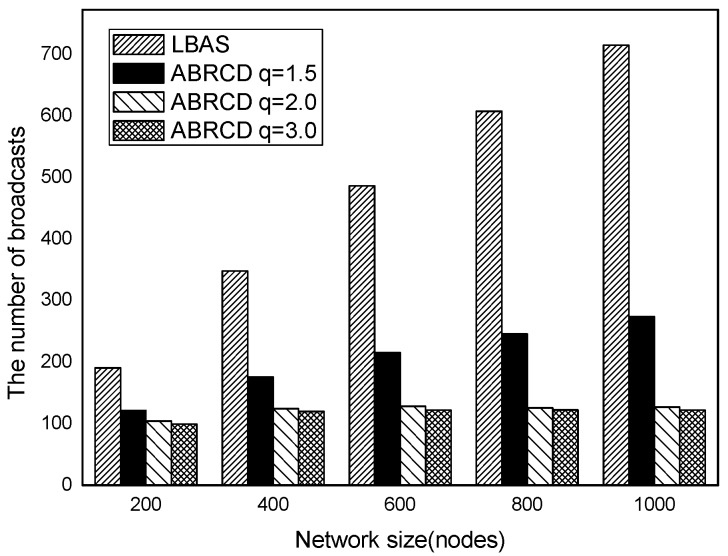
The impact of network size with fixed |*T*| = 120 on the number of broadcasts.

The impact of network size is shown in Figure 32, Figure 33, Figure 34, Figure 35, Figure 36 and Figure 37, with fixed |*T*| = 20, 40, 60, 80, 100, 120, respectively.

As is shown, ABRCD outperforms LBAS, and larger *q* performs better. The number of broadcasts is reduced up to 36.18~94.27%. Both schemes generate a larger number of broadcast when the network size is higher. This is obvious because every node chooses its time slot randomly, increasing the number of neighbors causes a covering node to need more covering time slots to transmit.

Moreover, with higher network size, the ratio of reduction of ABRCD over LBAS is higher. This is because when the network size is higher, the density of nodes is higher, nodes can reach more neighbors by enlarging radius. In addition, *q* = 2.0 and *q* = 3.0 almost reach the same low number of broadcasts for both the impact of |*T*| and network size. This shows that broadcasts reduction by enlarging the radius has a lowest bound.

### 6.2. Delay Analysis

Broadcast delay is calculated as Equations (5) and (6). $\varrho_{v}$ is from the output of Algorithm 1.

**Figure 38:**
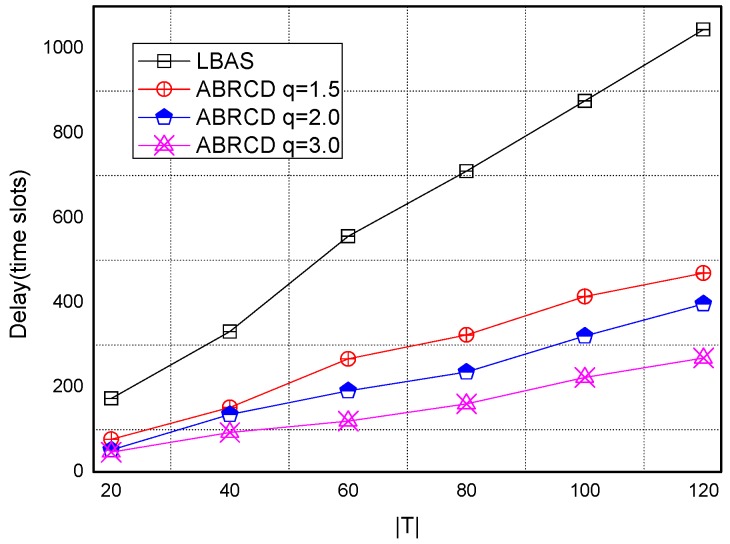
The impact of |*T*| with fixed network size = 200 on delay.

**Figure 39:**
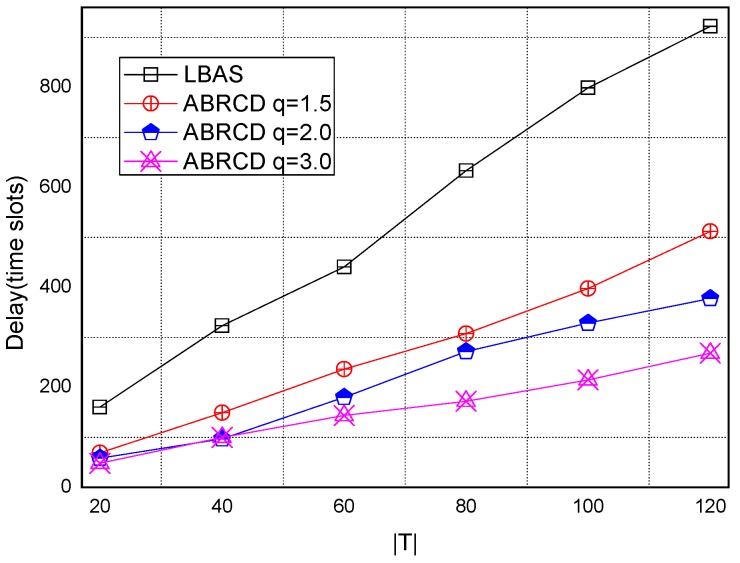
The impact of |*T*| with fixed network size = 400 on delay.

**Figure 40:**
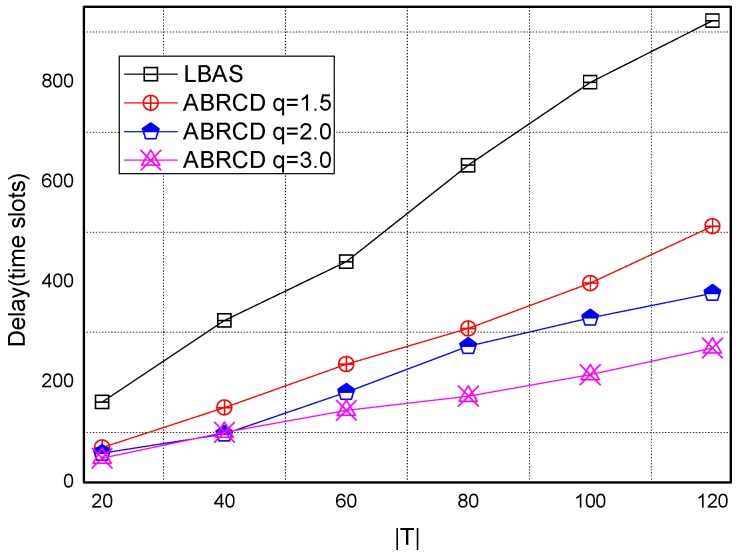
The impact of |*T*| with fixed network size = 600 on delay.

**Figure 41:**
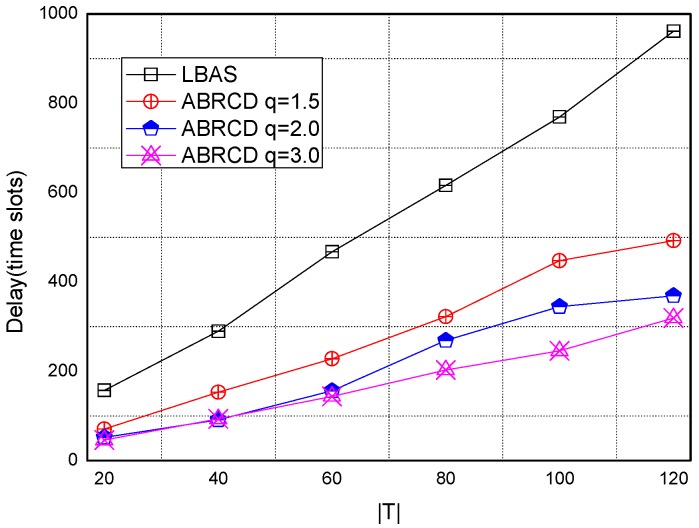
The impact of |*T*| with fixed network size = 800 on delay.

**Figure 42:**
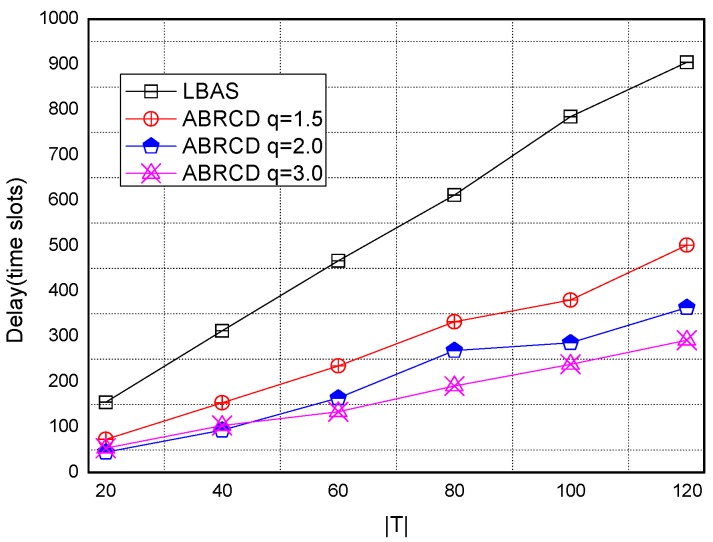
The impact of |*T*| with fixed network size = 1000 on delay.

Figure 38, Figure 39, Figure 40, Figure 41 and Figure 42 show the impact of |*T*| on broadcast delay. ABRCD outperforms LBAS, and generally larger *q* performs better. The broadcast delay is reduced by 41.11~78.42%, because reduction of transmissions leads to a decrement of delay and ABRCD can provide a broadcast tree with less depth. Both schemes generate higher delays with larger |*T*|. This is obvious, because increasing number of time slots makes it more probable for nodes to choose different active time slots, and a covering node needs to wait for more time until its neighbor is the active time slot.

**Figure 43:**
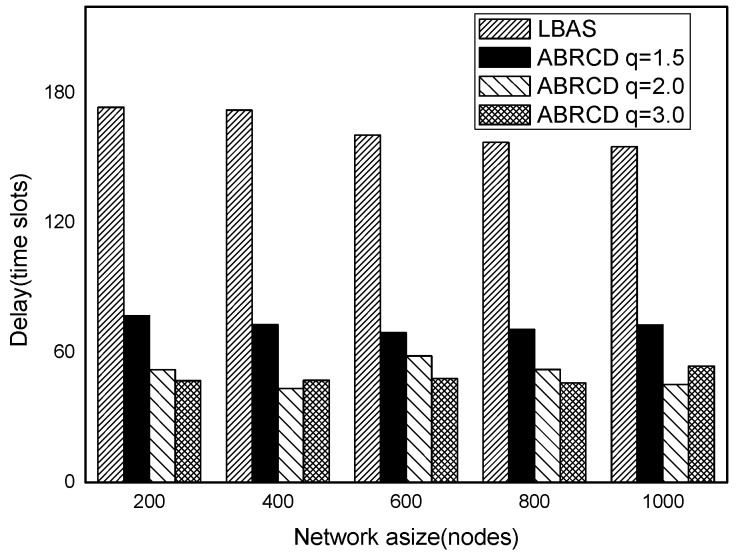
The impact of network size with fixed |*T*| = 20 on delay.

**Figure 44:**
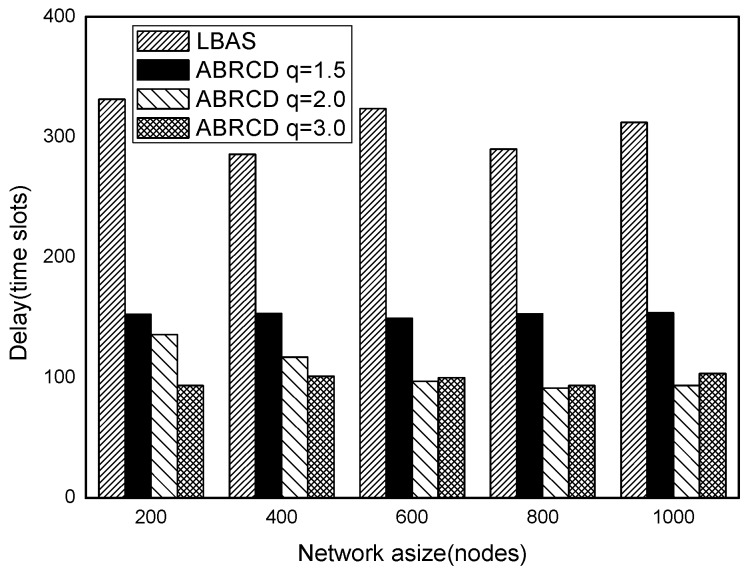
The impact of network size with fixed |*T*| = 40 on delay.

**Figure 45:**
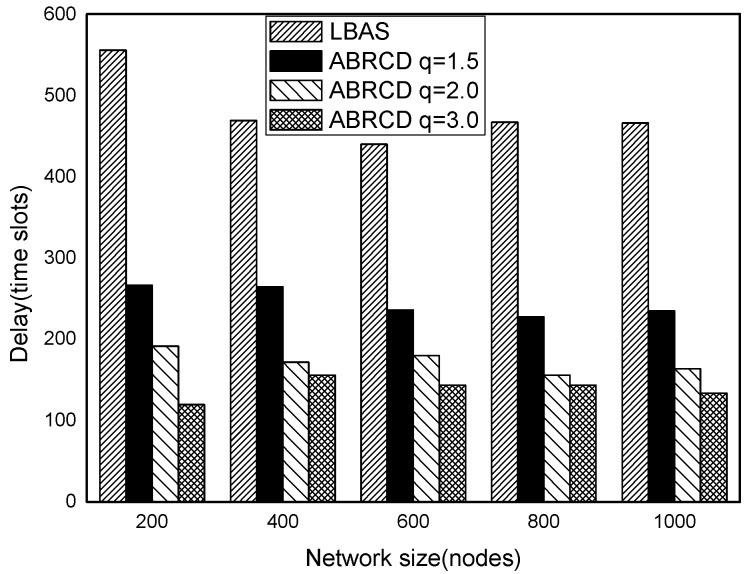
The impact of network size with fixed |*T*| = 60 on delay.

**Figure 46:**
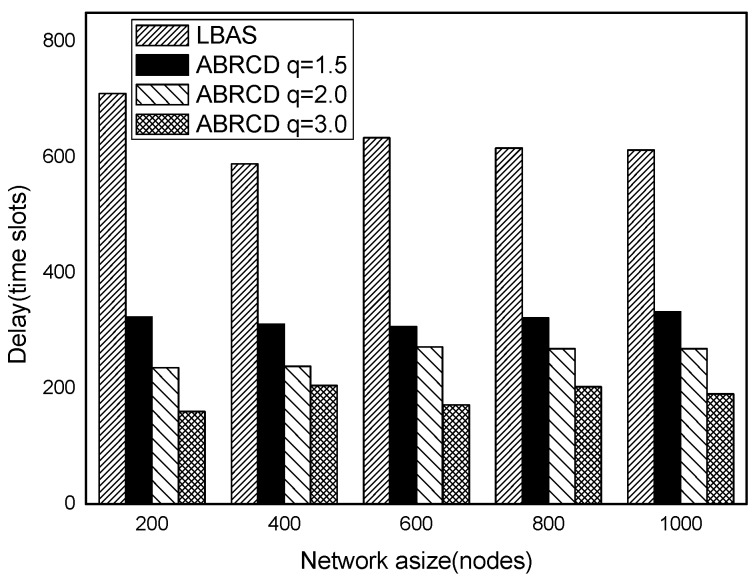
The impact of network size with fixed |*T*| = 80 on delay.

**Figure 47:**
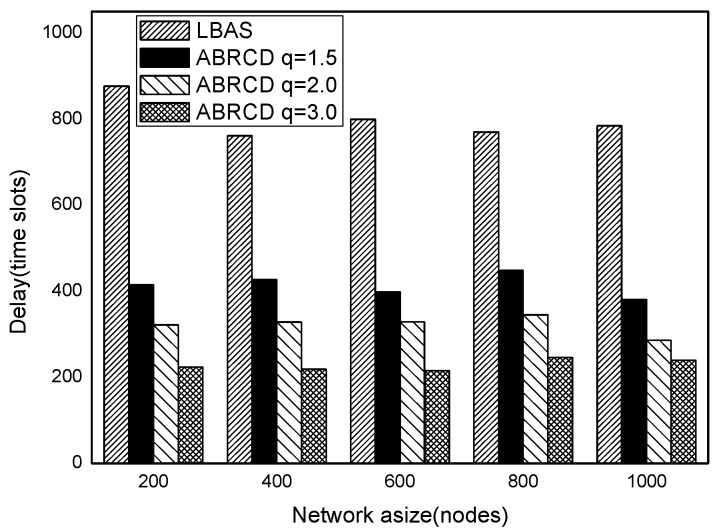
The impact of network size with fixed |*T*| = 100 on delay.

**Figure 48:**
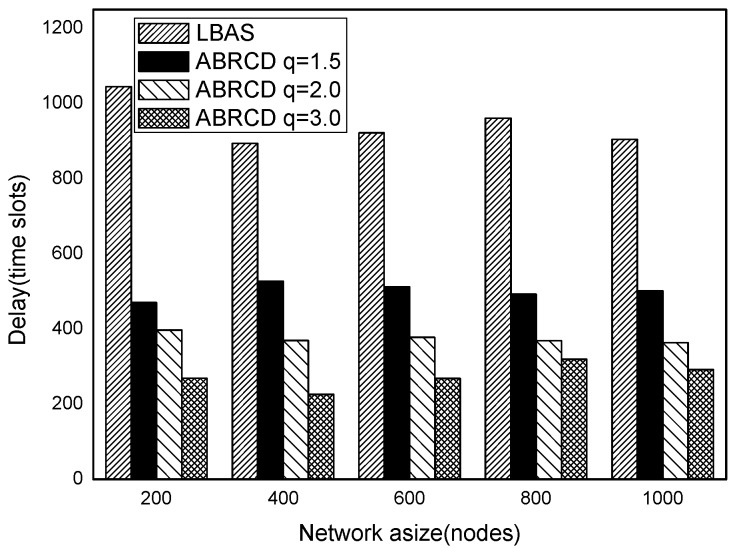
The impact of network size with fixed |*T*| = 120 on delay.

Figure 43, Figure 44, Figure 45, Figure 46, Figure 47 and Figure 48 show the impact of network size on broadcast delay. ABRCD outperforms LBAS, and generally larger *q* performs better. The broadcast delay is reduced by 41.11~78.42%, because reduction of transmissions leads to a decrement of delay and ABRCD can provide a broadcast tree with less depth. The delay of both the two schemes is not related with network size, because even though a high network size requires many transmissions, it brings high transmitting parallelism at the same time. Moreover, it can be observed that when network size is 200, the reduction ratio is always high, about 55% for *q* = 1.5, about 65% for *q* = 2.0, about 75% for *q* = 3.0 because when the network size is 200, the network is barely connected. Under these circumstances, enlarging the broadcast radius brings dominant benefits. Thus, ABRCD performs LBAS significantly when the network is sparse.

## 7. Conclusions

Software-defined wireless networks (SDWNs) bring great benefits for upgrading the services of sensors by updating software codes. This technology is very effective for sensor nodes deployed in the target area such as in environmental monitoring, industrial fields, smart fields and so on. Fast broadcasting of program codes to the entire network and energy conservation become important issues for SDWNs. Previous schemes provide optimization of minimum transmissions with fixed radius. The proposed Adaption Broadcast Radius-based Code Dissemination (ABRCD) scheme provides an idea of changing broadcast radius in geometric progression. Thanks to the benefits of a large radius at far areas that makes nodes to broadcast codes to many neighbors in one transmission and decrease data load at near area. ABRCD allows fast broadcasting and balancing of the energy consumption to increase the network lifetime at the same time. ABRCD can be used to modify many previous algorithms, bringing better performance to previous schemes.

For further study, Adaption Broadcast Radius based Code Dissemination (ABRCD) scheme and the Adjustable Duty Cycle Based Fast Disseminate (ADCFD) [25] scheme shall be combined to make a new scheme to achieve more significant performance improvements.

## Figures and Tables

**Table 1:** Parameters of the network.

Parameter	Value
Threshold distance ($d_{0}$) (m)	87
$E_{elec}$ (nJ/bit)	50
$\varepsilon_{fs}$ (pJ/bit/$m^{2}$)	10
$\varepsilon_{amp}$ (pJ/bit/$m^{4}$)	0.0013
Initial energy $E_{0}$ (J)	0.5

**Table 2:** Data packet dissemination using LBAS.

Period	Slot	Data Received at Node
1	0	
	1	$v_{1}$
	2	$v_{2}$
2	0	
	1	$v_{3}$
	2	$v_{4}$
3	0	$v_{5},v_{6}$
	1	
	2	$v_{7}$
4	0	$v_{8}$
	1	$v_{9},v_{10}$
	2	$v_{13}$
5	0	$v_{12},\;v_{14}$
	1	$v_{16}$
	2	$v_{11},\;v_{15}$

**Table 3:** Data packet dissemination using ABRCD.

Period	Slot	Data Received at Node
1	1	$v_{1}$
	2	$v_{2}$
2	0	
	1	
	2	$v_{4}$
3	0	$v_{7}$
	1	$v_{10}$
	2	$v_{3},\;v_{6},\;v_{13},v_{15},\;v_{16}$
4	0	$v_{5},\;v_{8},\;v_{11},v_{14}\;$
	1	$v_{9},\;v_{12}$
	2	
